# *O*-fucosylation stabilizes the TSR3 motif in thrombospondin-1 by interacting with nearby amino acids and protecting a disulfide bond

**DOI:** 10.1016/j.jbc.2022.102047

**Published:** 2022-05-18

**Authors:** Steven J. Berardinelli, Alexander Eletsky, Jessika Valero-González, Atsuko Ito, Rajashri Manjunath, Ramon Hurtado-Guerrero, James H. Prestegard, Robert J. Woods, Robert S. Haltiwanger

**Affiliations:** 1 Department of Biochemistry and Molecular Biology, Complex Carbohydrate Resource Center, University of Georgia, Athens, Georgia, USA; 2 Institute for Biocomputation and Physics of Complex Systems (BIFI), University of Zaragoza, Zaragoza, Spain; 3 Fundación Agencia Aragonesa para la Investigación y Desarrollo (ARAID), Zaragoza, Spain; 4 Department of Cellular and Molecular Medicine, Copenhagen Center for Glycomics, University of Copenhagen, Copenhagen, Denmark

**Keywords:** glycosylation, extracellular matrix, *O*-fucose, thrombospondin type 1 repeats, protein folding, CT, constant time, EGF, epidermal growth factor, ER, endoplasmic reticulum, HSQC, heteronuclear single quantum coherence, PTRPLS, Peters plus syndrome, TSP1, thrombospondin-1, TSR, thrombospondin type 1 repeat

## Abstract

Thrombospondin type-1 repeats (TSRs) are small protein motifs containing six conserved cysteines forming three disulfide bonds that can be modified with an *O*-linked fucose. Protein *O*-fucosyltransferase 2 (POFUT2) catalyzes the addition of *O*-fucose to TSRs containing the appropriate consensus sequence, and the *O*-fucose modification can be elongated to a Glucose-Fucose disaccharide with the addition of glucose by β3-glucosyltransferase (B3GLCT). Elimination of *Pofut2* in mice results in embryonic lethality in mice, highlighting the biological significance of *O*-fucose modification on TSRs. Knockout of *POFUT2* in HEK293T cells has been shown to cause complete or partial loss of secretion of many proteins containing *O*-fucosylated TSRs. In addition, POFUT2 is localized to the endoplasmic reticulum (ER) and only modifies folded TSRs, stabilizing their structures. These observations suggest that POFUT2 is involved in an ER quality control mechanism for TSR folding and that B3GLCT also participates in quality control by providing additional stabilization to TSRs. However, the mechanisms by which addition of these sugars result in stabilization are poorly understood. Here, we conducted molecular dynamics (MD) simulations and provide crystallographic and NMR evidence that the Glucose-Fucose disaccharide interacts with specific amino acids in the TSR3 domain in thrombospondin-1 that are within proximity to the *O*-fucosylation modification site resulting in protection of a nearby disulfide bond. We also show that mutation of these amino acids reduces the stabilizing effect of the sugars *in vitro*. These data provide mechanistic details regarding the importance of *O*-fucosylation and how it participates in quality control mechanisms inside the ER.

Thrombospondin type-1 repeats (TSRs^1^) are small, cysteine-rich protein motifs containing 50 to 60 amino acids. They contain six highly conserved cysteines that form three disulfide bonds which give TSRs a characteristic 3-dimensional fold and shape (1, 2, 3, 4, 5). TSRs that contain the consensus sequence C-X-X-S/T-C (first and second conserved cysteines for group 1 TSRs, second and third cysteines for group 2 TSRs) can be modified with an *O*-linked fucose (6, 7). The enzyme that adds fucose to TSRs is an endoplasmic reticulum (ER)–localized glycosyltransferase named Protein *O*-Fucosyltransferase 2 (POFUT2) (8). The *O*-fucose modification can further be extended to a disaccharide by β3-glucosyltransferase (B3GLCT) to form a glucoseβ1-3fucose disaccharide (9, 10). Additionally, TSRs are also frequently modified with another unusual sugar modification, *C*-mannosylation, at tryptophan residues when they reside in the specific consensus sequence of (W-X-X-W/C) (11, 12).

POFUT2 and B3GLCT both play important roles in development. Knockout of *Pofut2* in mice results in embryonic lethality during early development with severe gastrulation defects (13, 14). Mutations in *B3GLCT* result in Peters plus syndrome (PTRPLS), a congenital disorder of glycosylation (15, 16, 17, 18, 19). PTRPLS phenotypes are characterized by Peters anomaly of the eye (corneal opacity due to anterior segment dysgenesis), brachydactyly, craniofacial defects, and short stature. PTRPLS patients can also have varying additional congenital defects such as cleft lip/palate, genitourinary defects, hydrocephalus, developmental delay, and cardiac abnormalities (20). Global knockout of *B3glct* in mice recapitulates the bone growth phenotypes observed in PTRPLS patients, but zebrafish lacking B3GLCT enzyme activity develop normally (21, 22).

TSRs containing the POFUT2 consensus sequence occur in 49 cell-surface or secreted proteins which can contain a single or multiple tandem TSRs (23). Nearly half of these proteins belong to the A Disintegrin And Metalloproteinase with Thrombospondin Type-1 Repeats (ADAMTS) and ADAMTS-like extracellular matrix superfamily. Interestingly, elimination of individual ADAMTS proteins results in subsets of phenotypes that are observed in *Pofut2* or *B3glct*-null mice, or PTRPLS patients, conveying the importance of *O*-fucosylation of TSRs in ADAMTS proteins (14, 21). Other proteins containing TSRs that are POFUT2 and B3GLCT substrates are found in a variety of contexts that have diverse and substantial roles in biological processes (23) including properdin (involved in innate immunity) (3, 24, 25) and thrombospondin-1 (TSP1, involved in angiogenesis and ER stress) (26, 27, 28).

The function of *O*-fucosylation on TSRs has yet to be fully understood. One major observation is that POFUT2 serves as a folding sensor for TSRs during folding inside the ER (7, 29, 30). The ER is the folding compartment for proteins traversing though the secretory pathway, and both POFUT2 and B3GLCT are localized to the ER (8, 9, 10). POFUT2 only modifies folded TSRs, demonstrating that it can distinguish between folded and unfolded TSRs (31). The cocrystal structure of POFUT2 with a TSR also revealed how only folded TSRs fits into the POFUT2 active site, positioning the hydroxyl group of the serine or threonine to be modified precisely in the active site (6, 32). We have also shown that addition of fucose to a TSR stabilizes its folded state, making it much less susceptible to unfolding in a reducing environment *in vitro* (29). These results support the idea that *O*-fucosylation is involved in quality control for folding of TSRs inside the ER. In canonical ER quality control, the UDP-glucose:glycoprotein glucosyltransferase recognizes and adds a glucose to *N*-linked glycans of unfolded proteins, allowing them to interact with the chaperones calnexin and calreticulin (33). In contrast, POFUT2 recognizes folded TSRs and modifies them with fucose to lock the TSR into a stably folded structure allowing for proper secretion. Therefore, *O*-fucosylation by POFUT2 represents a noncanonical quality control pathway in the ER.

To further build on these observations, we hypothesized that *O*-fucose modifications stabilize TSRs in the folded state by interacting with side chains of underlying amino acids that are in close proximity to the *O*-fucose modification site. Using the third TSR (TSR3) from TSP1 as a model, we show here that the *O*-fucose modification does favorably interact with amino acids in close proximity and that mutating these residues results in a decrease in TSR3 stability. We propose that the *O*-fucose stabilizes folded TSRs by protecting the Cys^2^-Cys^6^ (C2-C6) disulfide bond, blocking it from reduction. This work provides a mechanism for how *O*-fucose stabilizes TSRs, conferring a quality control feature that leads to proper folding and secretion of POFUT2 substrates.

## Results

### MD simulations and crystal structure of *O*-fucosylated TSP1-TSRs suggest amino acids interacting with the *O*-fucose

**Figure 1:**
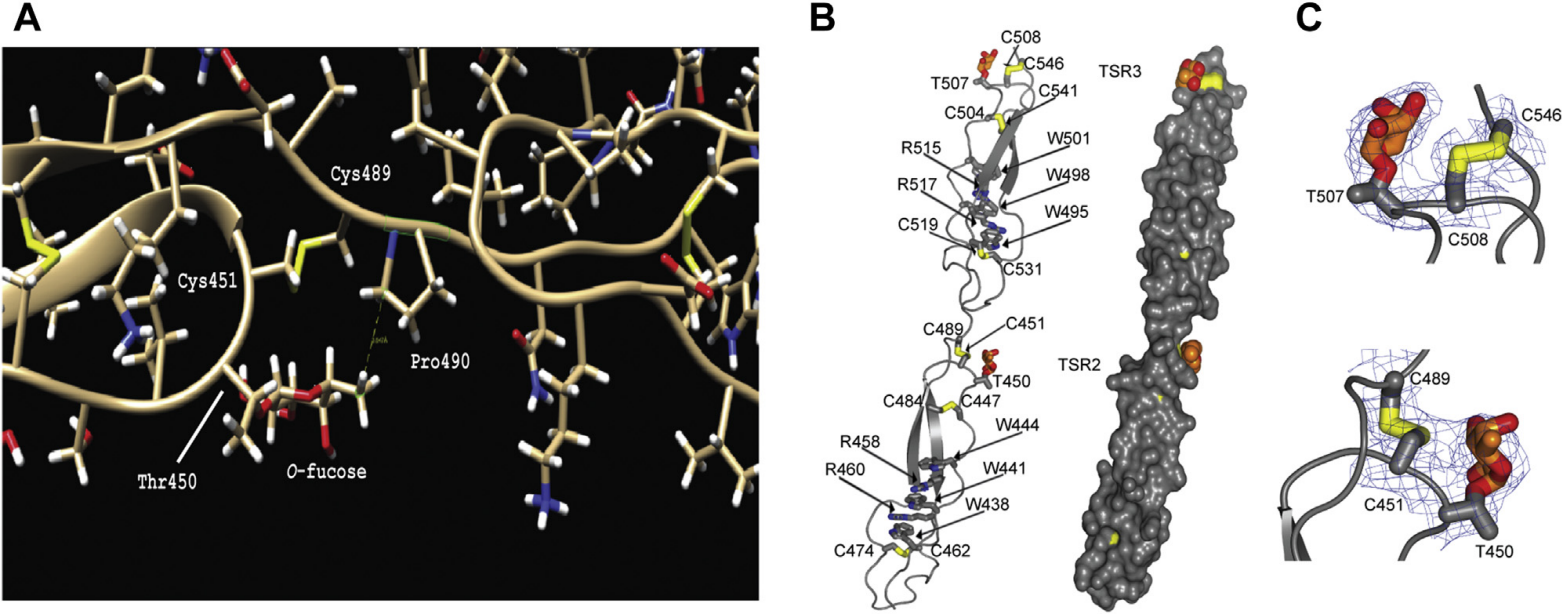
**MD simulations and x-ray crystallography reveal amino acids interacting with *O*-fucose on TSRs from TSP1.***A*, snapshot of a molecular dynamics (MD) simulation of TSR2 from TSP1. The MD simulation was performed by using the crystal structure coordinates from TSR2 (PDB = 1LSL) (2). The snapshot shows that Pro490 from TSR2 interacts with the C6 methyl from the fucose moiety around roughly 50% of the time during the trajectory. The hydrophobic face of the fucose is oriented toward the C2-C6 disulfide bond. Color coding: ribbon, *tan*; sulfur, *yellow*; nitrogen, *blue*; oxygen, *red*; hydrogen, *white*. *B*, a cartoon and space-filling model of *O*-fucosylated human TSP1 TSRs 1-3 crystal structure highlighting the close spatial proximity between the *O*-fucose and the C2-C6 disulfide bond for both TSRs. Only TSR2 and TSR3 are shown. Color coding: ribbon or space filling, *gray*; sulfur, *yellow*; oxygen, *red*; carbons of fucose, *orange*. *C*, hydrophobic interactions are observed between the hydrophobic face of the *O*-fucose on TSR2 and the C2-C6 disulfide bond (Cys451 and Cys489) from TSR2 and to a lesser extent, the C2-C6 disulfide bond (Cys508 and Cys546) from TSR3. Electron density maps are *2F*_O_–*F*_C_ (*blue*) contoured at 1σ for the C2-C6 disulfide bonds and the *O*-fucose moieties. Color coding: ribbon, *gray*; sulfur, *yellow*; oxygen, *red*; carbons of fucose, *orange*; electron density, *blue mesh*. TSP1, thrombospondin-1; TSR, thrombospondin type-1 repeat.

Previous studies from our laboratory had provided evidence that TSR3 from TSP1 modified with *O*-fucose monosaccharide (TSR3-Fuc) or the glucose-fucose disaccharide (TSR3-GlcFuc) provided stability to TSR3 making it less susceptible to unfolding under reducing conditions *in vitro* (29). Based on these findings, we hypothesized that both Fuc and GlcFuc provide stability by interacting with amino acids from the TSR that come into close spatial proximity with the *O*-fucose glycans. To determine which amino acids were being affected by the *O*-fucose, we first employed molecular dynamics (MD) simulations on *O*-fucosylated TSR2-TSR3 from TSP1 based on a previously published crystal structure (2). During the MD simulation, the C6 methyl group of the *O*-fucose residue on TSR2 interacted roughly 50% of the time with Pro490, located in the linker between the TSR2 and TSR3 domains (Fig. 1*A*). The *O*-fucose on TSR3 did not display any significant interactions with neighboring amino acids, which was not surprising given its location near the C-terminus of the TSR.

To identify additional interactions of the fucose with other amino acids, we crystallized the *O*-fucosylated form of TSRs 1-3 from human TSP1 and solved the structure at 2.6 Å (Table S1). The density for TSR2 and TSR3 was well defined, while no density was found for TSR1. Interestingly, we observed a continuous electron density between the fucose from TSR2 and the two cysteines (Cys451 and Cys489) that form the C2-C6 disulfide bond near the *O*-fucose site (Fig. 1, *B* and *C*). This electron density suggested a stabilizing hydrophobic interaction between the *O*-fucose atoms and the disulfide bond. The electron density between the *O*-fucose and disulfide bond from TSR3 was not continuous but the same hydrophobic interaction between the disulfide bridge and the *O*-fucose moiety was kept. In both cases, the hydroxyl groups of the fucose on TSR2 and TSR3 were pointing away from the disulfide bond and exposed to the solvent, allowing the more hydrophobic face of the *O*-fucose to interact with the C2-C6 disulfide bond. The same directionality of the hydroxyl groups was observed between the *O*-fucose and disulfide bond in TSR2 from the MD simulation described above (Fig. 1*A*). Interestingly, the *O*-fucose residues in the previously published structure (2) also covered the C2-C6 disulfide bonds, but the fucose was flipped. Although our MD simulation used the previously described crystal structure as the template, the orientation of the fucose moiety in both TSRs was the same as in Figure 1*B*, suggesting that the conformation of the fucose moiety in our crystal structure adopts the most stable orientation and is likely a more reliable conformation. These data suggest that the *O*-fucose is covering the disulfide bond, protecting it from being broken by oxidoreductases in the ER such as protein disulfide isomerase (34, 35).

### NMR reveals amino acids that are perturbed by the addition of *O*-fucose disaccharide on TSR3

We employed NMR spectroscopy to identify any additional amino acids that may be affected by *O*-fucose modification. We expressed TSR3 enriched with ^13^C and ^15^N isotopes to facilitate resonance assignment. Selected aliquots of purified ^13^C,^15^N-labeled TSR3 were modified with unlabeled *O*-fucose and *O*-fucose-glucose disaccharide *in vitro* (Table S2). Mass spectral analysis showed almost complete incorporation of ^13^C and ^15^N isotopes and complete modification of TSR3 by POFUT2 and B3GLCT (Fig. S1). Based on a set of 2D and 3D NMR spectra (Table S3), we obtained nearly complete resonance assignments (100.0% backbone, 95.5% side-chain, 97.5% overall) of ^1^H, ^13^C, and ^15^N spins of the native TSR3 segment (residues 490–548) in TSR3 and TSR3-Fuc. The only missing resonance assignments were those of Hβ, Hγ, Hδ, Cγ, and Cδ spins of Arg497 and Arg499. Since only 2D heteronuclear single quantum coherence (HSQC) spectra were used in resonance assignment of TSR-GlcFuc, the assignment completeness was lower (79.1% backbone, 76.1% side-chain, 77.4% overall); however, all backbone amide ^1^H and ^15^N spins have been assigned.

**Figure 2:**
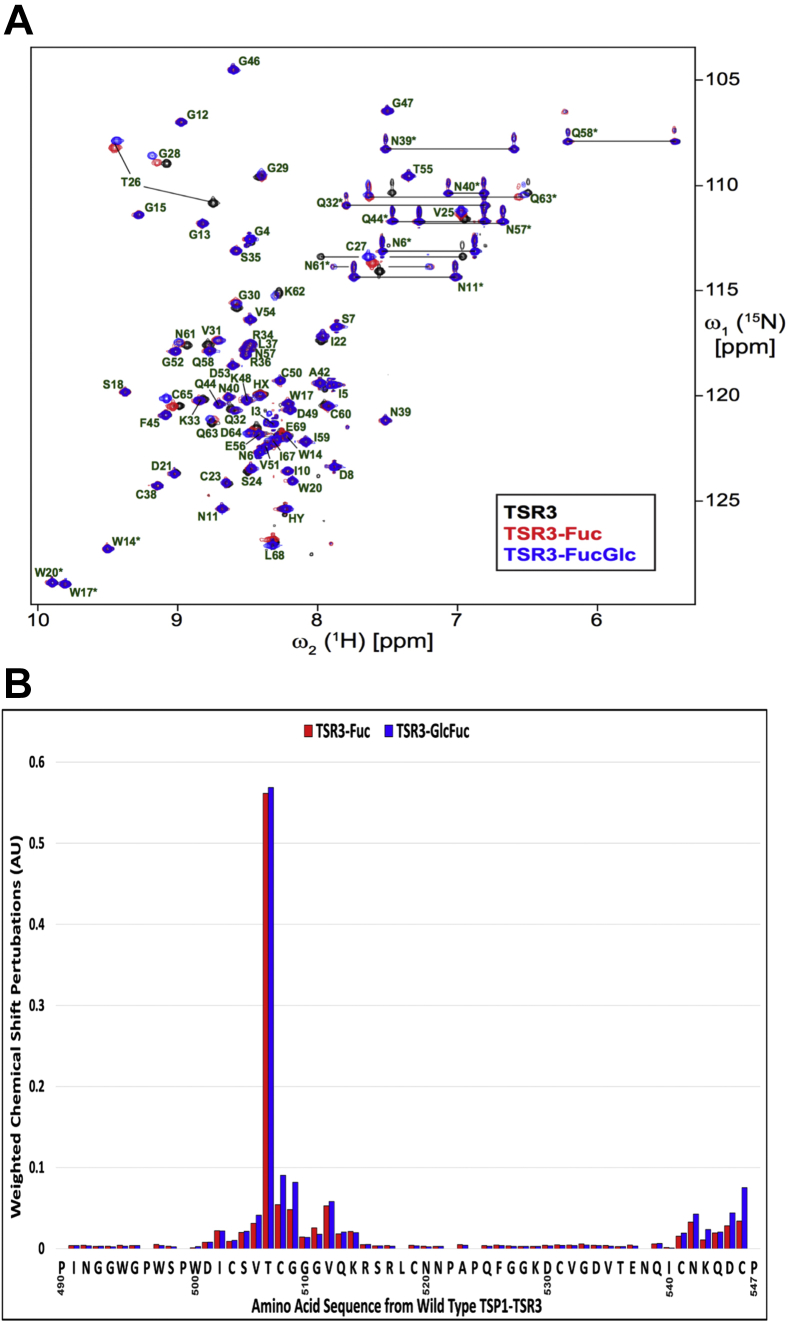
**Amino acids near the *O*-fucose disaccharide shows chemical shift perturbations upon addition sugars.***A*, 2D [^15^N, ^1^H] HSQC NMR spectra of ^13^C,^15^N-labeled unmodified TSR3 (*black*), TSR3-Fuc (*red*), and TSR3-GlcFuc (*blue*). Upon the addition of each *O*-fucose glycoform, large chemical shift perturbations were observed by amino acids that come into close proximity to the sugars. *B*, a graph of effective chemical shift perturbation Δδ of backbone amide ^1^H and ^15^N resonances for each TSR3 residue caused by addition of fucose (*red*) or fucose-glucose (*blue*) relative to unmodified TSR3 from the spectra shown in (*A*). The amino acid sequence of TSR3 is shown on the x-axis of the graph with chemical shift perturbation on the y-axis. HSQC, heteronuclear single quantum coherence; TSR, thrombospondin type-1 repeat.

Comparison of 2D [^15^N, ^1^H] HSQC spectra of TSR3 and TSR3-Fuc demonstrates significant amide chemical shift perturbations upon the covalent addition of fucose to TSR3 (Fig. 2*A*). As expected, the largest perturbation is observed for Thr507, the site of *O*-fucosylation. Interestingly, all other residues exhibiting large chemical shift perturbations were in close spatial proximity to the *O*-fucose. These include Ile503 and Val506 upstream of the *O*-fucose site, Gly509 and Val512 directly downstream to the O-fucose site along with Cys508, the second conserved cysteine located within the *O*-fucosylation consensus sequence (Fig. 2*B*). Interestingly, residues in the C-terminus, in close proximity to the *O*-fucose showed large perturbations as well. Of these, Asn542 showed a large shift along with Asp545 and Cys546, which form the conserved C2-C6 disulfide bond to Cys508 in TSR3. These large perturbations provide additional data suggesting that the *O*-fucose is protecting the C2-C6 disulfide bond.

Addition of glucose, to form TSR3-GlcFuc, further enhanced chemical shift perturbations for several residues (Fig. 2, *A* and *B*). Four amino acids directly upstream and downstream of the Thr507 *O*-fucose site such as Val506, Cys508, Gly509, and Val512 exhibited larger perturbations, while Ile503, Gly510, and Gly511 remained largely unchanged compared to TSR3-Fuc (Fig. 2, *A* and *B*). For the C-terminal residues Asn542, Lys543, Asp545, and Cys546, addition of glucose resulted in increased chemical shift perturbations compared to TSR3-Fuc (Fig. 2*B*). Taken together, these data identify residues affected by the addition of *O*-fucose mono- and di-saccharide modification on TSR3. This data also provides evidence that residues not in close proximity to the glycans did not have an effect on the chemical environment as their chemical shift perturbations were negligible.

**Figure 3:**
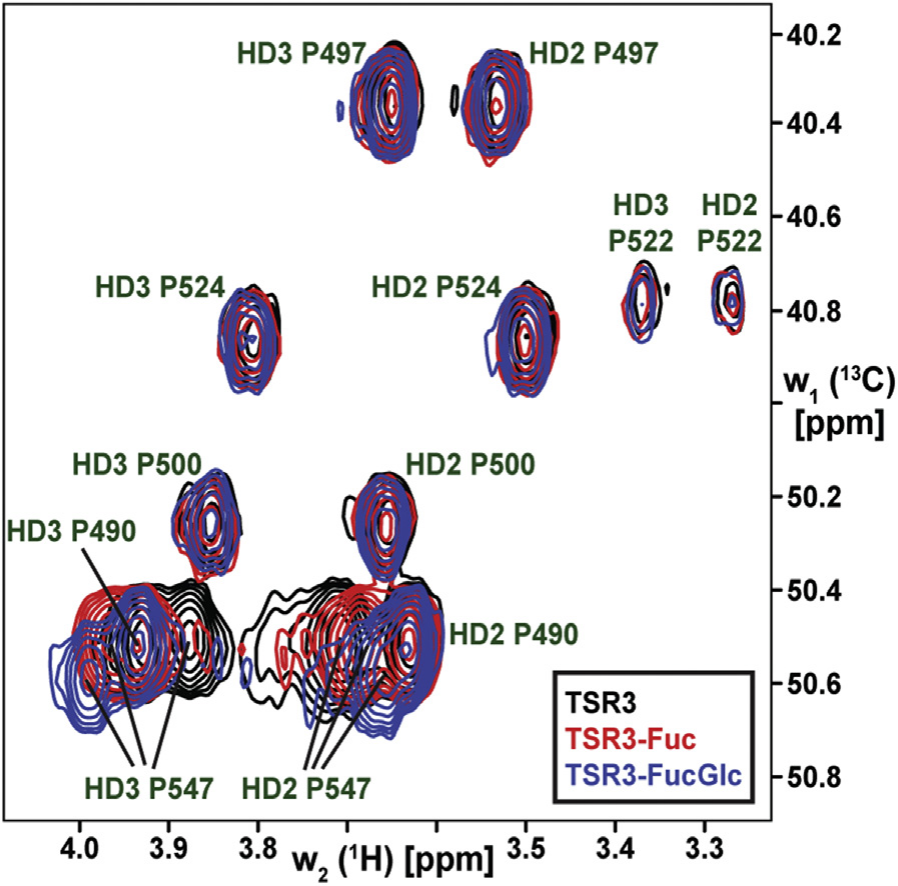
**The proline in the C6+1 position of TSR3 interacts with *O*-fucose and shows large chemical shift perturbations upon addition of sugars to TSR3.** Proline Hδ-Cδ region of 2D constant-time [^13^C,^1^H] HSQC NMR spectra of ^13^C,^15^N-labeled unmodified TSR3 (*black*), TSR3-Fuc (*red*), and TSR3-GlcFuc (*blue*). Upon the addition of each sugar, large chemical shift perturbations were observed for the Pro547 at the C6+1 position of TSR3, but not for the other prolines. HSQC, heteronuclear single quantum coherence; TSR, thrombospondin type-1 repeat.

Pro490 is immediately C-terminal to the sixth conserved cysteine (C6+1) of TSR2 and showed an interaction with the methyl group of fucose in the MD simulations in Figure 1*A*. Pro547 is in the equivalent C6+1 position in TSR3. Since proline is an imino acid, it was not detected in the 2D [^15^N, ^1^H] HSQC experiment in Figure 2*A* and thus not represented in the effective chemical shift perturbation plot of Figure 2*B*. However, the effects of addition of the *O*-fucose mono- or di-saccharide can be observed in 2D aliphatic constant-time [^13^C, ^1^H] HSQC spectrum (Fig. 3). HD2, HD3, and CD resonances of Pro547 were noticeably perturbed upon the addition of fucose and glucose, but other prolines that are not close to the *O*-fucose site in TSR3 remained unchanged (Fig. 3). Furthermore, analysis of ^13^C-edited [^1^H,^1^H] NOESY spectrum recorded for TSR3-Fuc with ^13^C-labeled O-fucose demonstrated the presence of NOE cross-peaks between H4 and H6 resonances of O-fucose, and HD2, HD3, and HG resonances of Pro547 (Fig. S2). This represents direct evidence of the corresponding atoms within TSR3-Fuc being in spatial proximity (<5–6 Å) in solution.

Importantly, prolines are commonly found in C6+1 position in many TSRs, implying a potential structural importance for prolines at this particular position in the C-terminus (Table 1). For example, two of the three TSRs from TSP1 and all four TSRs from properdin with consensus sequences for *O*-fucose modification have a proline in the C6+1 position. ADAMTS13, which is highly important for blood coagulation and blood clotting, has five out of eight TSRs with a proline in the C6+1 position as well. Additionally, 65% of all the TSRs with the consensus sequence for *O*-fucosylation from the ADAMTS superfamily have a proline in the C6+1 position (Table S4). Taken together, these data suggest an important interaction between prolines in the C6+1 position and the *O*-fucose modification on TSRs.

**Table 1:** Prolines are highly conserved at the C6+1 position in TSRs

POFUT2 substrates	TSRs w/Consensus	TSRs w/Consensus and Pro@C6+1 position	Percentage of TSRs w/Consensus and Pro@C6+1 position
TSP1	3/3	2/3	66
Properdin	4/7	4/4	100
ADAMTS-1	3/3	2/3	66
ADAMTS-2	3/4	3/3	100
ADAMTS-3	3/4	1/3	33
ADAMTS-4	1/1	1/1	100
ADAMTS-5	2/2	1/2	50
ADAMTS-6	3/5	3/5	60
ADAMTS-7	6/8	3/6	50
ADAMTS-8	2/2	2/2	100
ADAMTS-9	12/15	6/12	50
ADAMTS-10	3/5	3/3	100
ADAMTS-12	6/8	2/6	33
ADAMTS-13	7/8	5/7	71
ADAMTS-14	3/4	2/3	66
ADAMTS-15	3/3	2/3	66
ADAMTS-16	6/6	5/6	83
ADAMTS-17	4/5	1/4	25
ADAMTS-18	4/5	3/5	60
ADAMTS-19	4/5	1/4	25
ADAMTS-20	11/15	6/11	55
Total	93/118	58/93	65

The table shows the number of times prolines occur at the C6+1 position from all TSRs found in ADAMTS proteins, TSP1, and properdin. From these TSRs, 65% have a proline C6+1 position. Summarized from Table S4.

### Mutation of residues perturbed by *O*-fucose glycans reduces the ability of the glycans to stabilize folded TSR3

**Figure 4:**
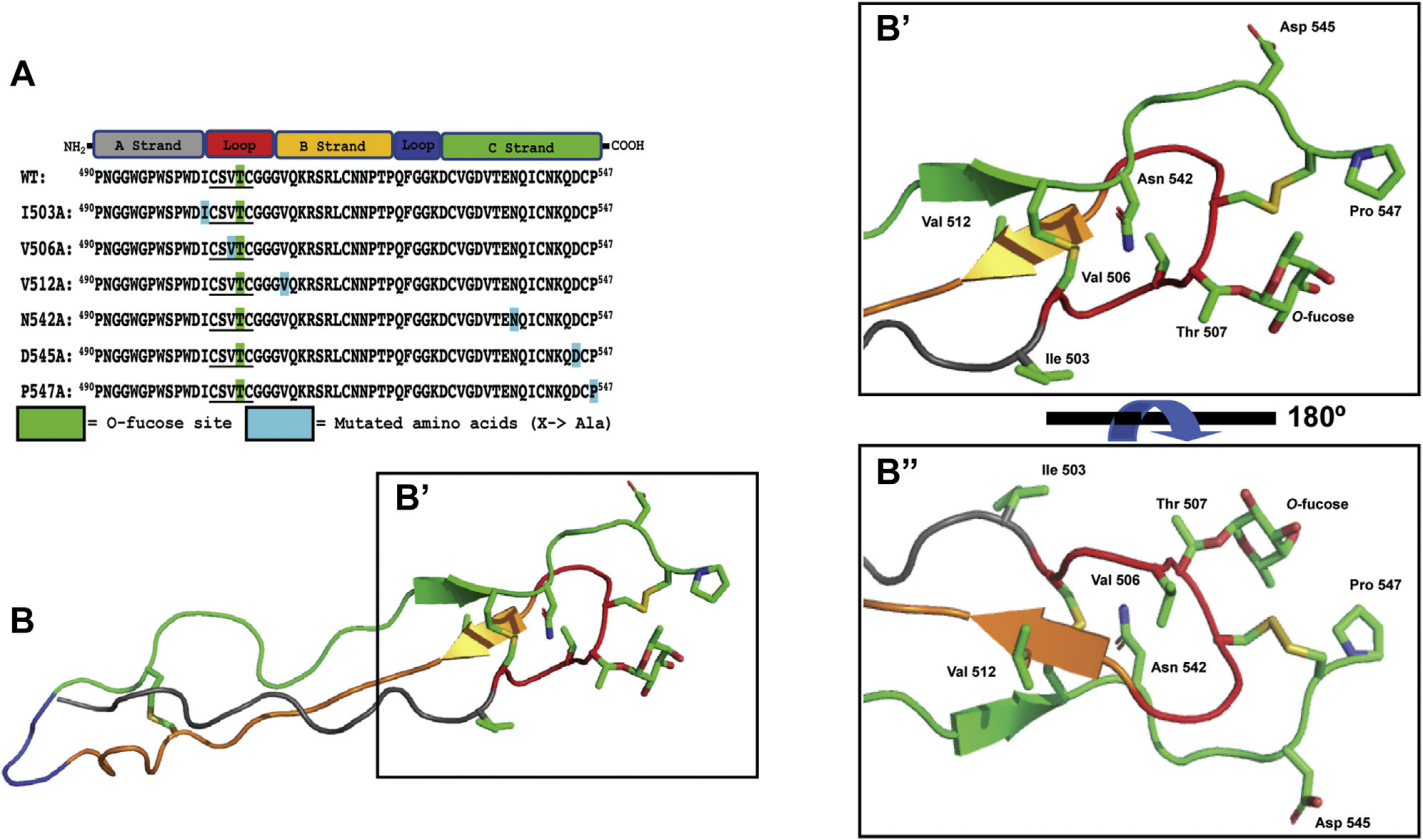
**Mutation of amino acids that could affect *O*-fucose stabilization and their relative proximities to the *O*-fucose.***A*, a domain map highlighting the secondary structures/domains from TSR3. The amino acid sequence from WT TSR3 and mutant constructs are displayed, and amino acids mutated to alanine are highlighted in *blue*. The underlined amino acids correspond to the POFUT2 consensus sequence, and the threonine highlighted in *green* corresponds to the *O*-fucose site on TSR3. *B*, *B′*, and *B′′*, cartoon representation of TSR3 generated from the crystal structure from Figure 1*B* highlighting the amino acids being mutated (in *sticks*) and their relative proximity to the *O*-fucose. Coloring of A, B, and C-strands is as in *A*. *B′* is a zoomed in version of *B* and *B′′* is flipped 180° to aid in the visualization of the proximity of mutated amino acids to the *O*-fucose. TSR, thrombospondin type-1 repeat.

To determine if the *O*-fucose-interacting and glycan-perturbed residues identified in the experiments above influence *O*-fucose stabilization on folded TSR3, we engineered single alanine point mutations to several of these residues into TSR3 (Fig. 4*A*), expressed the mutated TSRs in *Escherichia coli*, and incubated with POFUT2 and B3GLCT to create all three glycoforms: unmodified TSR3, TSR3-Fuc, and TSR3-GlcFuc. Although all TSR3 mutants were shown to be poorer substrates for POFUT2 than WT TSR3 (Fig. S3), full modification of TSR3-Fuc and TSR3-GlcFuc after overnight incubation with POFUT2 and B3GLCT were confirmed by nano-LC-MS (Fig. S4). Some amino acids that showed large perturbations upon addition of *O*-fucose (*i.e.*, Cys508, Gly509, Cys546) were not tested due to either poor protein expression or disruption of disulfide bond formation upon mutation (Table S5). Importantly, all amino acids that were tested come into close proximity to the *O*-fucose site (Fig. 4, *B*, *B′* and *B′′*).

**Figure 5:**
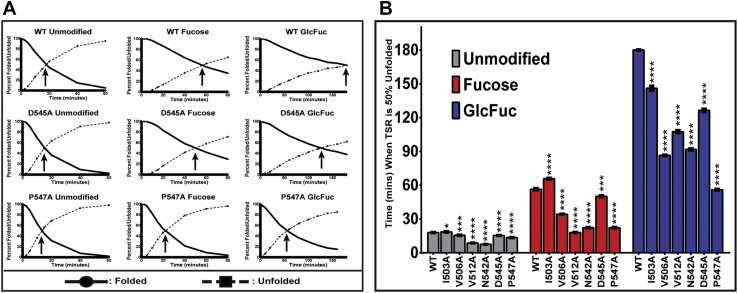
**TSR3 mutants decrease the ability of *O*-fucose glycans to stabilize folded TSRs.***A*, plots of the reductive unfolding rate of WT TSR3, and the P547A and D545A mutants. Each TSR3 was unfolded with no modification (unmodified), TSR3-Fuc modification, and TSR3-GlcFuc modification. The *black arrow* indicates the inflection point of (when 50% of the TSR is folded/unfolded) of each glycoform. When comparing these three TSRs, P547A had a large decrease in stability whereas D545A had a minor but substantial decrease in stability when compared to WT TSR3. *B*, inflection points for the average of triplicate assays of WT and all TSR3 mutants and all glycoforms of each: unmodified (*gray*), TSR3-Fuc (*red*), and TSR3-GlcFuc (*blue*). A one-way ANOVA test was used to determine statistical significance (∗*p* < 0.05, ∗∗∗*p* < 0.001 and ∗∗∗∗*p* < 0.0001). TSR, thrombospondin type-1 repeat.

To test whether these mutations affected the ability of *O*-fucose mono- or di-saccharide to stabilize TSR3, we performed reductive unfolding assays as described in Experimental procedures. When subjected to unfolding conditions (0.75 mM DTT, 37 °C), the unmodified WT TSR3 reached an inflection point (when 50% of the TSR3 is folded/unfolded) at roughly 18 min (Fig. 5*A*, top left). Upon addition of the glycans, WT TSR3-Fuc and TSR3-GlcFuc significantly increased the stability and inflection point to roughly 60 and 180 min, respectively (Fig. 5*A*, top middle, top right). These results highlight the stabilizing effect *O*-fucosylation has on WT TSR3 which was previously determined in our lab under similar conditions (29). Repeating these experiments on mutated TSRs revealed the stability was decreased to varying degrees for most residues mutated (Fig. 5, *A* and *B*). When unmodified TSRs were unfolded, there was a minimal effect on the stability when comparing WT to all mutants (Fig. 5*B*). However, the stability was significantly reduced in most cases for TSR3-Fuc and TSR3-GlcFuc modified mutants (Fig. 5*B*).

The P547A mutation showed a large decrease in stability with inflection points reduced to 30 and 60 min for TSR3-Fuc and TSR3-GlcFuc, respectively. Large reductions in stability were also seen with the V506A, V512A, N542A mutants. Each of these mutants had roughly a two-fold (or more) reduction in stability provided by both TSR3-Fuc and TSR3-GlcFuc modifications when compared to WT TSR3. The D545A mutation had a slight decrease in stability provided by TSR3-Fuc modification and roughly a 33% decrease provided by the TSR3-GlcFuc modification. The only mutation to not have a decrease in stability in the context of TSR3-Fuc modification was the I503A mutation. However, a modest reduction in stabilization was observed upon I503A TSR3-GlcFuc modification (Fig. 5*B*). This could be because Ile503 is further from the *O*-fucose site compared to the other residues mutated (Fig. 4, *B′* and *B′′*). Overall, these results suggest that *O*-fucose mono- and di-saccharides stabilize TSR3 by interacting with side chains of underlying amino acids.

## Discussion

In these studies, we sought to understand how addition of *O*-fucose glycans stabilize TSRs in the presence of reducing agents. Using MD simulations, X-ray crystallography, and NMR, we showed that the *O*-fucose covers the C2-C6 disulfide bond of TSR3, protecting it from reduction. The orientation of the *O*-fucose is mediated by interactions with the side chains of several underlying amino acids. In particular, a proline at the C6+1 position plays an important role, and database searches reveal that prolines in this position are conserved in many TSRs with POFUT2 consensus sites. We also have direct evidence of an interaction between *O*-fucose and proline in the form of NOE peaks, and the involvement of the H6 methyl of *O*-fucose indicates that this interaction is likely hydrophobic in nature. Mutation of most of the underlying amino acids to alanine, including the proline, reduced the ability of fucose or the fucose-glucose disaccharide to stabilize TSR3 under reducing conditions. These results strongly suggest that the *O*-fucose glycans stabilize TSR3 by interacting with several underlying amino acids, resulting in protection of the C2-C6 bond from reduction.

**Figure 6:**
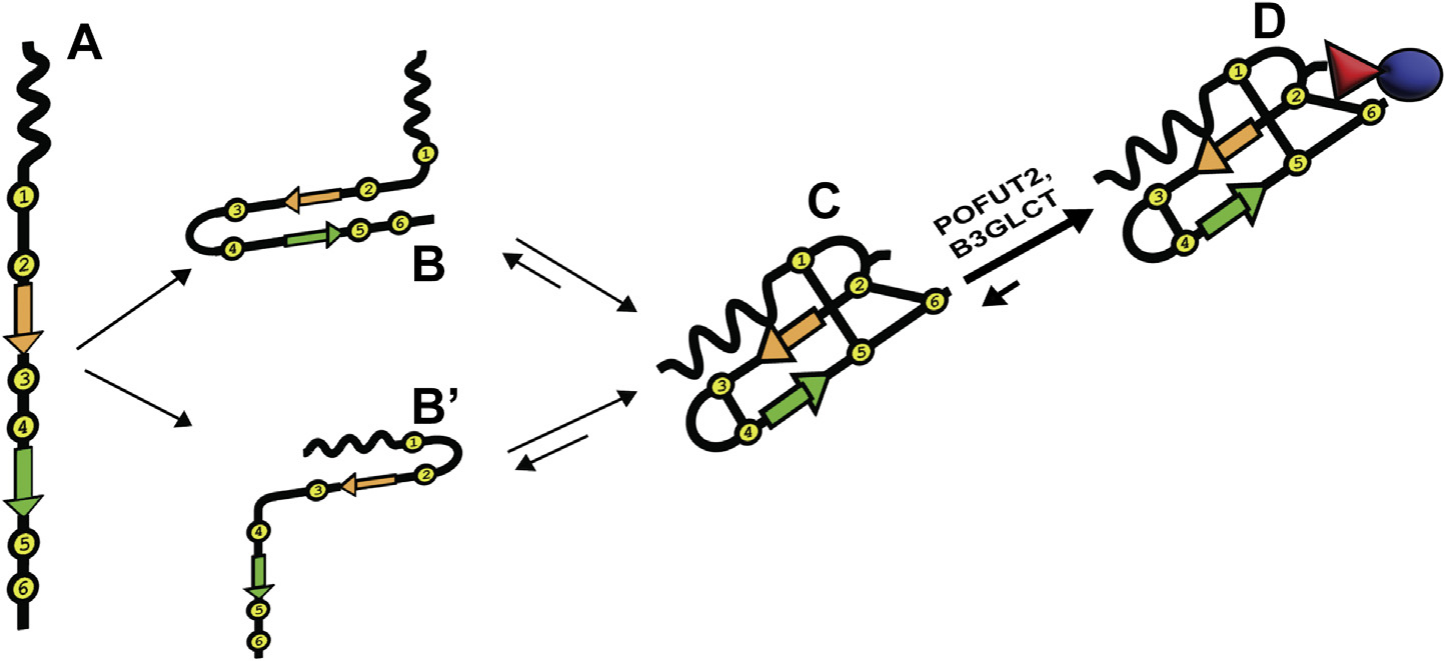
**A model for how increased stability by *O*-fucose assists in folding of TSRs.** As the TSR is translated and extruded into the lumen of the ER in an unfolded state (*A*). A-, B- and C-strands are colored as in Figure 4. The TSR then begins to fold, likely driven by the association of anti-parallel β-strands in the B- and C-strands (*B*) or by stacking of Trp residues in the A-strand with Arg residues in the B-strand (*B′*), following formation of the folded TSR and disulfide bond formation (*C*). Note that this structure would still be in equilibrium with more unfolded versions of the TSR due to the presence of oxidoreductases in the ER that can make and break disulfide bonds. Not shown are potential mispaired disulfide bonds either within the TSR or with other free cysteines in the local environment. Once the correctly folded form of the TSR exists, it will fit into the active site of POFUT2 (6), leading to addition of *O*-fucose, stabilizing the folded TSR and protecting the C2-C6 bond. B3GLCT, also in the ER, will rapidly add a glucose to the *O*-fucose, providing further stabilization (*D*). Addition of the sugars will shift the folding equilibrium toward the *O*-fucose modified product. Since TSRs frequently occur in tandem, this process is repeated further, driving the folding equilibrium toward *O*-fucose modified product for each TSR. Color coding: cysteines, *yellow circles*; fucose, *red triangle*; glucose, *blue circle*. ER, endoplasmic reticulum; TSR, thrombospondin type-1 repeat.

Our results also provide additional evidence that POFUT2 and B3GLCT function in a noncanonical quality control pathway for folding of TSRs in the ER (7, 29). In the crowded environment of the ER where disulfide bonds are formed, a quality control check is needed for independent domains like TSRs, especially in proteins where they are tandemly repeated. For instance, ADAMTS20 has 15 TSRs, containing 90 cysteines just in the TSRs that need to find their partners. ADAMTS20 secretion is lost in *POFUT2*-null or *B3GLCT*-null HEK293T cells (21), suggesting that folding of ADAMTS20 TSRs is dependent on the addition of both fucose and glucose. We propose that POFUT2 recognizes the correctly folded form of a single TSR and modifies it with *O*-fucose, stabilizing it by protecting the C2-C6 disulfide bond (Fig. 6). Addition of glucose by the ER-localized B3GLCT provides additional stability. These modifications would prevent ER oxidoreductases from breaking this bond, which would cause the TSR to reenter a folding cycle, delaying folding and increasing the possibility of aberrant disulfide bond formation. This model helps to explain why most POFUT2 substrate proteins are either not secreted, or secretion is significantly reduced, from *POFUT2*-null HEK293T cells (14, 21, 36, 37). The model is also consistent with our data showing that *in vitro* folding of TSR3 is accelerated in the presence of POFUT2 and GDP-fucose, but not in the presence of POFUT2 alone (29). Addition of the *O*-fucose is required. The fact that secretion of only some POFUT2 substrates, such as ADAMTS20, are reduced in *B3GLCT*-null HEK293T cells (21, 37) suggests that some TSRs are more difficult to fold *in vivo*, and the presence of the glucose is necessary for stabilization, folding, and secretion of these TSRs.

*C*-mannosylation of TSRs has also been shown to have similar stabilizing effects on TSRs (38). However, most TSRs in the ADAMTS/L superfamily have the minimal (W-X-X-C) consensus sequence compared to the extended (W-X-X-W-X-X-W-X-X-C) consensus sequence. In the extended consensus sequence, all tryptophans can be modified, although the final tryptophan in the extended consensus sequence is modified at much lower stoichiometries than the first two tryptophans (11). To date, our group and others have shown that mass spectral site mapping of TSRs containing the minimal (W-X-X-C) consensus sequence often have little to no *C*-mannose modification leaving *O*-fucosylation as the major glycan modification–driving stabilization on these TSRs (21, 36, 39, 40). The importance of *O*-fucose modification on ADAMTS/L TSRs may be more important for folding, quality control, and the secretion and proper function than TSR-containing proteins modified with *C*-mannose. However, recently, we observed the secretion of ADAMTS17 in the limb buds of mice where *Pofut2* has been ablated using *Prrx1-Cre* (41), suggesting loss of ADAMTS/L secretion may be cell-type specific. This could be due to cell-specific expression of chaperones that help TSRs to fold in the absence of POFUT2, or of the four *C*-mannosyltransferases that have been identified (11), one of which (DPY19L3) is capable of modifying W-X-X-C sites.

Recent data suggests that *O*-fucosylation on TSRs is not only important for folding and secretion but has functions in the extracellular environment as well. A recent study showed that an *O*-fucose on TSR3 of brain-specific angiogenesis inhibitor 1 (BAI1) is in direct contact with its ligand, reticulon-4 receptor (RTN4R) (42). BAI–RTN4R interactions control dendritic arborization, axon elongation, and synapse formation. Mutation of the *O*-fucose site, or elimination of *POFUT2* in cells, blocks BAI1-RTN4R binding. This is highly reminiscent of *O*-fucose residues on the epidermal growth factor-like (EGF) repeats of the Notch receptor, which is added by the POFUT2 homolog, POFUT1 (7). POFUT1 is also ER localized and only modifies properly folded EGF repeats (43). We have shown that like TSRs, *O*-fucosylation by POFUT1 stabilizes EGF structure, and cell-surface NOTCH1 expression is reduced in *POFUT1*-null HEK293T cells (44). In addition, the *O*-fucose residue on EGF12 of NOTCH1 is in direct contact with Notch ligands Delta-like 4 and Jagged1 (45, 46). Mutation of the *O*-fucose site on EGF12 significantly reduces NOTCH1 function (14, 46, 47). Thus, *O*-fucose modifications on both TSRs and EGF repeats appear to function in protein folding in the ER and regulating protein–protein interactions in the extracellular spaces.

## Experimental procedures

### TSP1 TSR2-3 protein structure used for MD simulations

A crystal structure of two repeating units of the thrombospondin-1 type 1 (PDB code 1LSL) was obtained from the PDB server (rcsb.org). The structure contained two β-linked fucose residues attached at Thr 432 and 489 and six disulfide bonds, which were defined using the TLEaP module of AmberTools (48). Prior to solvation, a single chloride ion was added using TLEaP to neutralize the overall charge on the system. Analysis focused on *O*-fucose of TSR2 because the *O*-fucose on TSR3 was too close to the C-terminus in the structure.

### Energy minimization and MD simulations

The fucosylated structure was placed in a periodic box of 22,916 TIP3P water molecules (49) with a 12 Å buffer between the glycoprotein and the box edge. Energy minimization of all atoms was performed for 10,000 steps of steepest decent under constant volume conditions. MD was subsequently performed under nPT conditions (1 atm, 300 K) with the CUDA implementation of the PMEMD (50, 51) simulation code, as present in the Amber14 software suite (52). The GLYCAM06j force field (53) and Amber14SB force field (54) were employed for the carbohydrate and protein moieties, respectively. A Berendsen barostat with a time constant of 1 ps was employed for pressure regulation, while a Langevin thermostat with a collision frequency of 2 ps^−1^ was employed for temperature regulation. A nonbonded interaction cut-off of 8 Å was employed. Long-range electrostatics were treated with the particle-mesh Ewald method (55). Covalent bonds involving hydrogen were constrained with the SHAKE algorithm (56), allowing an integration time step of 2 fs to be employed. The energy minimized coordinates were heated to 300 K over 40 ps with restraints on the Cα atoms of the protein to permit the water molecules to reorient, before being equilibrated for 2 ns at 300 K, with no restraints. Following equilibration, production simulations were performed at 300 K for 100 ns. Snapshots were collected at 10 ps intervals.

### Cloning, expression, and purification of human TSP1-TSRs 1-3 and CePoFUT2 double mutant for crystallization

The cloning, expression, and purification of human TSR1-2-3 (*Hs*TSR1-2-3) from human thrombospondin 1 and *Ce*PoFUT2 double mutant R298K-R299K were previously described (6).

### Fucosylation of human TSP1-TSRs 1-3 and mass spectrometry

Human TSP1-TSRs 1-3 (∼10 mgs) were expressed in *E*. *coli* and fucosylated *in vitro* by adding 700 μM GDP-Fuc and 700 μM *Ce*PoFUT2 double mutant in a final volume of ∼2 ml at 18 °C overnight. The reaction was performed in buffer 25 mM Tris–HCl, pH 7.5, 0.15 M NaCl. The fucosylated TSP1-TSRs 1-3 was further purified by using 1 × 5 ml HiTrap Q FF column. The fucosylated TSP1-TSRs 1-3 was recovered from the flow-through since it did not bind to the column while the *Ce*PoFUT2 double mutant bound to the column.

The fucosylation of TSP1-TSRs 1-3 was confirmed by MALDI-TOF mass spectrometry. While the mass of the nonfucosylated TSP1-TSRs 1-3 was 19294.562 Da, the mass for the fucosylated protein was 19728.308 Da rendering a difference of 433.74 Da, which corresponds closely to the addition of three fucose moieties.

### Crystallization of O-fucosylated TSP1-TSRs 1-3

Crystals of the fucosylated TSP1-TSRs 1-3 were obtained by mixing 0.5 μl of protein solution (a mix formed by the fucosylated TSP1-TSRs 1-3 in 25 mM Tris–HCl, pH 7.5, 0.15 M NaCl) with 0.5 μl of precipitant solution (10–15% PEG 8000, 200 mM zinc acetate in 0.1 M MES pH 6.5) against 60 μl of precipitant solution. The crystals were obtained by sitting drop vapor diffusion at 18 °C. The crystals were cryoprotected in mother liquor containing 20% glycerol and flash frozen in liquid nitrogen.

### Structure determination and refinement

Data were collected in the beamline I03 of DLS at a wavelength of 0.97 Å. The data were processed and scaled using the XDS package (57) and CCP4 (58) software. Relevant statistics are given in Table S1. The crystal structure was solved by molecular replacement with Phaser (58) and using human TSP1-TSRs 1-3 (PDB entry 1LSL) as the template. Initial phases were further improved by cycles of manual model building in Coot (59) and refinement with REFMAC5 (60). The final model was validated with PROCHECK; model statistics are given in Table S1. The asymmetric unit of the crystal contains one molecule of the *Hs*TSR1-2-3, and only density for TSR2 and TSR3 was well defined in agreement with a previous report (2). The Ramachandran plot shows that 78.4% and 21.6% of the amino acids are in most favored and allowed regions, respectively.

### Recombinant expression of ^15^N,^13^C-labeled TSR3 glycoforms for NMR spectroscopy

TSR3 from human TSP1 was expressed in BL21(DE3) cells transformed with the construct pET20b+-hTSP1-TSR3 (31). The cells were cultured in supplemented minimal growth medium as previously described (61). The medium was supplemented with 0.05 mg/ml thiamin (Fisher BioReagents), 0.01 mg/ml biotin (Fisher BioReagents), 10 mM iron chloride (SIGMA), 10 mM copper sulfate (SIGMA), 10 mM manganese chloride (II) (SIGMA), and 10 mM zinc sulfate (SIGMA) (all reagents were sterile filtered). For ^15^N and ^13^C isotopic labeling, [^15^N]-ammonium chloride (Cambridge Isotope Laboratories) and [U^13^C]-D-glucose (Cambridge Isotope Laboratories) were added to the medium at 0.1% and 0.2% final concentration, respectively. Cultures were grown in a 37 °C shaking incubator until reaching an absorbance at 600 nm (A600) of 0.6∼0.8. Protein expression was induced by addition of IPTG to a concentration of 0.4 mM. After adding IPTG, the culture was incubated 12 to 16 h at 20 °C with shaking. Cells were collected by centrifugation, resuspended in 50 mM Tris–HCl, pH 8.0, 1 mM PMSF, and lysed by sonication using 10 s bursts and repeated ten times with 2-min intervals between each burst. The lysate was clarified by centrifugation, and TSR3 was purified using Ni-NTA agarose (GBiosciences). The column was rinsed with ten column volumes of wash buffer consisting of 0.5 M NaCl, 10 mM imidazole in 10 mM Tris–HCl, pH 7.5, 0.15 M NaCl (tris-buffered saline), and the protein was eluted with 250 mM imidazole in tris-buffered saline. To separate folded TSR3 from misfolded variants, C18 reverse-phase HPLC was performed as described previously (31). GDP-[U13C]Fucose was synthesized as previously described from GTP and [U13C]-L-Fucose (Cambridge Isotope Laboratories) (62). TSR3-Fuc was synthesized by incubation of 20 mM [^15^N, ^13^C]-TSR3 with 200 mM natural abundance GDP-Fucose (Carbosynth) or GDP-[U13C]Fucose and *Caenorhabditis elegans* POFUT2 (6) in buffer containing 50 mM Hepes, pH 6.8, 10 mM manganese chloride, overnight at 37 °C. TSR3-GlcFuc was generated in a similar reaction in addition to recombinant human B3GLCT (63) and 200 mM UDP-Glucose (SIGMA). The sugar-modified TSR3 proteins, TSR3-Fuc and TSR3-GlcFuc, were repurified from the reaction mixture using C18 reverse-phase HPLC and lyophilized. Efficient modification of TSR3 with fucose and glucose was confirmed by nanoLC-MS using a C4 reverse-phase column attached to a Q-Exactive Plus Orbitrap mass spectrometer (Thermo Fisher Scientific) before NMR analysis. To prepare NMR samples (Table S2), all TSR3 glycoforms (TSR3, TSR3-Fuc, and TSR3-GlcFuc) were resuspended in 20 mM sodium phosphate buffer, pH 6.5, 100 mM NaCl, 4 μM 4,4-dimethyl-4-silapentane-1-sulfonic acid, 0.02% sodium azide, and 7% D2O.

### 2D-HSQC NMR experiments

NMR spectra of TSR3 glycoforms (Table S3) were acquired at 25 °C using Agilent DD2 800 and 900 MHz instruments with inverse detected ^1^H(^13^C,^15^N) 5 mm cryogenic probes, Varian VNMRS 600 MHz instruments with inverse detected ^1^H(^13^C,^15^N) 3 mm and 5 mm cryogenic probes, and Bruker AVANCE NEO 900 MHz instrument with inverse detected ^1^H(^13^C,^15^N) 5 mm room-temperature TXI probe or ^1^H(^13^C,^15^N) 5 mm cryogenic ^13^C,^15^N(^1^H) TCO probe. Constant time (CT) delays in 2D aliphatic and aromatic [^13^C, ^1^H] CT-HSQC experiments were set to 26 and 16 ms, respectively. Mixing time in ^13^C/^15^N-edited NOESY spectra was set to 100 ms. Uniformly sampled NMR data from Bruker and Varian/Agilent instruments were Fourier transformed using TopSpin (Bruker BioSpin) or NMRPipe (64), respectively. Nonuniformly sampled NMR experiments utilized Poisson-gap sampling and were reconstructed using hmsIST (65) followed by Fourier transformation with NMRPipe. ^1^H, ^13^C, and ^15^N chemical shifts were referenced relative to 4,4-dimethyl-4-silapentane-1-sulfonic acid with ^13^C and ^15^N shifts referenced indirectly *via* gyromagnetic ratios. Visualization and analysis of NMR spectra, peak picking, and NMR resonance assignment were performed using CARA (66). Assignment of backbone ^1^H, ^15^N, ^13^CO, ^13^C^α^, and ^13^C^β^ resonances of nonglycosylated TSR3 was initially generated automatically with AutoAssign (67) and then interactively validated and finalized within CARA. Side-chain resonances were assigned based on 3D HBHA(CO)NH, 3D (H)CCH-COSY, 3D (H)CCH-TOCSY, and 3D ^15^N/^13^C-edited NOESY spectra. Assignment of backbone and side chain resonances of TSR3-Fuc was performed interactively using CARA based on TSR3 assignments. Resonance assignments of ^1^H and ^13^C spins of the covalently linked fucose were obtained from 3D (H)CCH-COSY, 3D (H)CCH-TOCSY, and 3D ^15^N/^13^C-edited NOESY spectra. Assignments of ^1^H, ^13^C, and ^15^N resonances of TSR3-GlcFuc were obtained by comparing 2D [^15^N, ^1^H] HSQC and aliphatic 2D [^13^C, ^1^H] CT-HSQC spectra of TSR3-Fuc and TSR3-GlcFuc. Effective backbone amide chemical shift perturbation values were calculated according to the formula $\Delta \delta =\sqrt{\frac{1}{2}\left(\Delta \delta_{H}^{2}+{\left(0.14\Delta \delta_{N}\right)}^{2}\right)}$, where Δ*δ*_*H*_ and Δ*δ*_*N*_ are perturbation values in ppm of ^1^H and ^15^N spins respectively.

### Design, expression, and glycan modification of WT and mutant TSR3

All point mutations were performed using the construct pET20b+-hTSP1-TSR3 (31) as a template with primers pairs for each point mutation (Table S4). For amplification of mutants, 4 ng of template plasmid were incubated with 1% glycerol, 0.5 μM of forward and reverse primers, and 1× of CloneAmpHifi PCR premix (Takara) in a total volume of 20 μl. Thirty cycles of amplification were performed using 98 °C for denaturing and 58 °C for annealing for each mutant. After confirming proper point mutations by sequencing, each construct was expressed, purified, and modified in the same fashion as described in the previous section above but with natural abundance instead of ^13^C,^15^N isotope labeling. Lyophilized samples were then resuspended in a buffer consisting of 50 mM Tris–HCl, pH 8.0, 0.2% NaN3, and 2 μM of beta-mercaptoethanol.

### Reductive unfolding assays of WT and mutant TSRs

Unmodified TSR3, TSR3-Fuc, and TSR3-GlcFuc TSRs (WT and mutants, 20 μM) were unfolded at 37 °C using 0.75 mM DTT in 100 mM Tris–HCl, pH 8.0. At multiple timepoints, unfolding was stopped by acid-quenching using a final concentration of 2% TFA as described previously (29, 68). Samples were then spin filtered using a 0.2 μm centrifugal filter (VWR) and injected on an HPLC equipped with an analytical C18 column for separation of folded and unfolded peaks. A 30-min gradient (10–80% acetonitrile in 0.1% TFA) was employed to separate folded and unfolded TSRs. Folded and unfolded peaks were quantified by area under the curve using UV quantitation at 214 nm then converted to percentages and graphed using Prism software. Each mutant was analyzed in triplicate assays, and error bars shown were generated by SD and statistical analysis was performed using a one-way ANOVA test.

### POFUT2 enzymatic assays

POFUT2 assays were performed using GDP-Glo assays (Promega) to quantify transfer of fucose to mutant and WT TSR3. For each assay, 8 ng of POFUT2 was incubated with increasing amounts of mutant or WT TSR3 and 160 μM of Ultra-pure GDP-fucose (Promega). Samples were incubated at 37 °C for 20 min and the reaction was quenched by adding equal volumes of Nucleotide Detection Reagent which is provided in the GDP-Glo assay kit. Samples were then incubated in the dark at room temperature for 1 h. Luminescence of free GDP was then read using a Cytation 3 and quantified based on a standard GDP curve. Units were converted to pmol/min/mg and graphed and analyzed to a nonlinear substate inhibition fit using Prism software.

## Data availability

The crystal structure of the fucosylated TSP1-TSRs 1-3 was deposited at the RCSB PDB with accession code 7YYK. Assigned chemical shifts, HSQC peak lists, and time-domain NMR data for TSR3, TSR3-Fuc, and TSR3-GlcFuc have been deposited in the BioMagResBank with accession numbers 51351, 51356, and 51358, respectively.

## Supporting information

This article contains supporting information.

## Conflict of interest

The authors declare that they have no conflicts of interest with the contents of this article.
