# Supplementary material for: O-fucosylation stabilizes the TSR3 motif in thrombospondin-1 by interacting with nearby amino acids and protecting a disulfide bond
Source: J Biol Chem. 2022 May 18;298(6):102047. doi: 10.1016/j.jbc.2022.102047 (PMC9198472; doi:10.1016/j.jbc.2022.102047)
Supplement: Supplemental Tables S1–S5 and Figures S1–S4 [file mmc1.pdf]

Supporting Information for:

O-Fucosylation stabilizes the TSR3 motif in thrombospondin-1 by interacting with nearby amino acids and protecting a disulfide bond

Steven J. Berardinelli, Alexander Eletsky, Jessika Valero-González, Atsuko Ito, Rajashri Manjunath, Ramon Hurtado-Guerrero, James H. Prestegard, Robert J. Woods, and Robert S. Haltiwanger

Contents:

Tables S1-S5

Figures S1-S4

	O-fucosylated TSR1-2-3 form from human thrombospondin 1
**Data collection**	
Space group	$C222_1$
Cell dimensions	
a, b, c (Å)	66.15, 85.20, 62.12
α, β, γ (°)	90, 90, 90
Resolution (Å)	20-2.60 (2.74-2.60*)
R_{merge}	0.197 (0.859)
R_{pim}	0.073 (0.317)
$I / \sigma I$	6.2 (2.7)
Completeness (%)	99.8 (100)
Redundancy	8.3 (8.2)
Mn(I) half-set correlation CC(1/2)	0.987 (0.871)
**Refinement**	
Resolution (Å)	2.60
No. reflections	46810
R_{work} / R_{free}	0.249/0.316
No. atoms	
Protein	856
Fucose	22
Ethylenglycol	4
Waters	22
B-factors (Å^2)	
Protein	70.55
Fucose	86.95
Ethylenglycol	82.9
Waters $^+$	60.73
R.m.s. deviations	
Bond lengths (Å)	0.0127
Bond angles (°)	1.7379

Table S1: Data collection and refinement statistics for *O*-fucosylated hTSP1-TSR1-3. A list of data collection parameters and refinement statistics for the crystal structure shown in Fig.1B. One crystal was used to determine the crystal structure. *Values in parentheses are for the highest-resolution shell.

Name	Isotope Labeling	Tube size	Vol. [ul]	Conc. [uM]
TSR3NC	[U-^{13}C,^{15}N]-TSR3	3 mm	90	720
TSR3NC-Fuc	[U-^{13}C,^{15}N]-TSR3, [U-^{13}C]-Fuc	3 mm	90	260
TSR3NC2	[U-^{13}C,^{15}N]-TSR3	3 mm	96	360
TSR3NC2-Fuc	[U-^{13}C,^{15}N]-TSR3	5 mm	300	240
TSR3NC2-GlcFuc	[U-^{13}C,^{15}N]-TSR3	5 mm	300	240

Table S2: Isotope labeled TSR3 samples for NMR spectroscopy

Sample	NMR Spectrum	B_0 [MHz][a)]	Probe type[b)]
TSR3NC	2D [^{15}N, ^{1}H] HSQC	800 V	5mm CR
	2D [^{13}C, ^{1}H] CT-HSQC ali	800 V	5mm CR
	2D [^{13}C, ^{1}H] CT-HSQC aro	800 V	5mm CR
	3D HNCO (NUS)[c)]	600 V	5mm CR
	3D (HACA)CONH	600 V	5mm CR
	3D CBCA(CO)NH	600 V	5mm CR
	3D HNCACB	600 V	3mm CR
	3D HBHA(CO)NH	600 V	5mm CR
	3D (H)CCH-COSY ali	800 V	5mm CR
	3D (H)CCH-TOCSY ali	800 V	5mm CR
	3D (H)CCH-COSY aro	900 V	5mm CR
	3D ^{15}N/^{13}C-edited NOESY	800 V	5mm CR
	3D ^{15}N-edited NOESY	800 V	5mm CR
TSR3NC2	2D [^{15}N, ^{1}H] HSQC	900 B	5mm RT
	2D [^{13}C, ^{1}H] CT-HSQC ali	900 B	5mm CR
TSR3NC-Fuc	2D [^{15}N, ^{1}H] HSQC	900 V	5mm CR
	2D [^{13}C, ^{1}H] CT-HSQC ali	900 V	5mm CR
	2D [^{13}C, ^{1}H] CT-HSQC aro	900 V	5mm CR
	3D HNCO (NUS 6.25%)[c)]	900 V	5mm CR
	3D (HACA)CONH	900 V	5mm CR
	3D CBCA(CO)NH (NUS 25%)[c)]	900 V	5mm CR
	3D HNCACB	900 V	5mm CR
	3D HBHA(CO)NH	600 V	5mm CR
	3D (H)CCH-COSY ali	900 V	5mm CR
	3D (H)CCH-TOCSY ali	900 V	5mm CR
	3D (H)CCH-COSY aro	900 V	5mm CR
	3D ^{15}N/^{13}C-edited NOESY	800 V	5mm CR
TSR3NC3-Fuc	2D [^{15}N, ^{1}H] HSQC	900 B	5mm RT
	2D [^{13}C, ^{1}H] CT-HSQC ali	900 B	5mm CR
TSR3NC3-FucGlc	2D [^{15}N, ^{1}H] HSQC	900 B	5mm RT
	2D [^{13}C, ^{1}H] CT-HSQC ali	900 B	5mm CR

Table S3: 2D and 3D NMR spectra used in resonance assignment of TSR3 glycoforms and chemical shift perturbation analysis. a) V-Varian/Agilent: B-Bruker b) RT-room-temperature probe; CR-cryogenic probe c) Non-uniform sampling.

Protein	TSR No.	C5-C6 Sequence	Protein	TSR No.	C5-C6 Sequence	Protein	TSR No.	C5-C6 Sequence
ADAMTS1	TSR1	CNLEDCP	ADAMTS9	TSR6	CELPSCH	ADAMTS15	TSR3	CVLRPC-*
	TSR2	CADHPCP		TSR7	CSVTPCG			
	TSR3	CTMAECS		TSR8	CSMSPCP	ADAMTS16	TSR1	CNSQKCP
				TSR9	CESGPCP		TSR2	CKVSACP
ADAMTS2	TSR1	CSRQDCP		TSR10	CNTHACP*		TSR3	CNSQSCP
	TSR2	CNPQECS*		TSR11	CRGGRCP		TSR4	CLLQRCH
	TSR3	CSRELCP		TSR12	CQGPRCP		TSR5	CAPLPCP
	TSR4	CRLGPCP		TSR13	CSLQPCE		TSR6	CNTHFCP
				TSR14	CYLRDCP			
ADAMTS3	TSR1	CNTEECQ		TSR15	CRNVYNCE*	ADAMTS17	TSR1	CENLPCP
	TSR2	CNIQECT*					TSR2	CNLHPCQ*
	TSR3	CSRELCP	ADAMTS10	TSR1	CNTDDCP		TSR3	CEGQDCL
	TSR4	CQLPPCN		TSR2	CNTDDCP*		TSR4	CEDYSGCY*
				TSR3	CHGPTCP		TSR5	CYQEVCN
ADAMTS4	TSR1	CNTEDCP		TSR4	CNLRRCP			
				TSR5	CEAKCD-*	ADAMTS18	TSR1	CNINPCN
ADAMTS5#	TSR1	CSLMPCP					TSR2	CNSHACP*
	TSR2	CLLKKC-*	ADAMTS12	TSR1	CNVHPCR		TSR3	CVLGRCP
				TSR2	CHEKACP		TSR4	CNRRACP
ADAMTS6	TSR1	CNTDPCP		TSR3	CNRDILCP*		TSR5	CNTNFCP
	TSR2	CNTEPCP		TSR4	CGLQQCP			
	TSR3	CNNQSCP		TSR5	CHLRPCA	ADAMTS19	TSR1	CENPPCP
	TSR4	CSLGRCP*		TSR6	CNPEPCE*		TSR2	CNEQPCQ
	TSR5	CESKCDS*		TSR7	CNEHLCC		TSR3	CEGQDCM
				TSR8	CNQQACK		TSR4	CEDYSKCY*
ADAMTS7	TSR1	CNLQACP					TSR5	CHLQPCN
	TSR2	CSEQPCP	ADAMTS13	TSR1	CNTQACE			
	TSR3	CNRHVPCP*		TSR2	CVLEPCP	ADAMTS20	TSR1	CNTDSCP
	TSR4	CSLPLCR		TSR3	CNPQPCP		TSR2	CNTDCE-*
	TSR5	CHLRPCA		TSR4	CVGMSCP*		TSR3	CHGNCV-*
	TSR6	CGAQPCL		TSR5	CQAVPCP		TSR4	CNEFSCP
	TSR7	CNTHPCT*		TSR6	CSLEPCP		TSR5	CELHTCA
	TSR8	CGTEDCE*		TSR7	CLIADCT		TSR6	CVLTPCS
				TSR8	CWAGPCV		TSR7	CFTPCG-*
ADAMTS8	TSR1	CHTEECP					TSR8	CSLAACP
	TSR2	CESQLCP	ADAMTS14	TSR1	CNSEECP		TSR9	CGPGPCP
				TSR2	CNQHPCS*		TSR10	CHMHACP
ADAMTS9	TSR1	CNTEPCL		TSR3	CLRVPCP		TSR11	CRSVRCP
	TSR2	CGTDCD-*		TSR4	CSLPACG		TSR12	CWSQDCV
	TSR3	CSGECN-*					TSR13	CRNPPCN
	TSR4	CSEFPCP	ADAMTS15	TSR1	CNLEPCP		TSR14	CINSC-*
	TSR5	CQQPECA		TSR2	CGEPCP-*		TSR15	CYANDCK

Table S4: List of sequences between C5 and C6 of all TSRs from the ADAMTS superfamily. Table 1 was created using all the TSRs from human ADAMTS proteins in the above table except for TSRs labeled with an asterisk. TSRs with an asterisk were not used because a) they did not have a predicted consensus sequence for *O*-fucosylation, b) they had more than four amino acids between C5 and C6, c) they had less than four amino acids between C5 and C6, or d) if the C6 of the TSR terminated a protein sequence. ADAMTS5 is denoted with a hashtag (#) indicating that ADAMTS5 and ADAMTS11 are the same proteins which is why ADAMTS11 is not listed in the table.

TSR3 Mutation	Primer	Sequence (5'→3)
I503A	Forward	ATGGGACgcCTGTTCTGTCACCTGTGGA
	Reverse	AGAACAGgcGTCCCATGGTGACCAAGGA
V506A	Forward	TCTGTTCTGcCACCTGTGGAGGAGGGGTA
	Reverse	CCACAGGTGgCAGAACAGATGTCCCATGGT
V512A	Forward	GGAGGAGGGGcACAGAAACGTAGTCGTCTCTGCAAC
	Reverse	ACGTTTCTGTgCCCCTCCTCACAGGTGACAGAACA
N542A	Forward	AGATCTGCgcCAAGCAGGACTGTCCAATTCTCGAGC
	Reverse	CCTGCTTGgcGCAGATCTGGTTTTCTGTTACATCAC
D545A	Forward	GAAAACCAGATCTGCAACAAGCAGGcgTGTCCAATTCTCGAGCA
	Reverse	GAATTGGACAcgCCTGCTTGTTGCAGATCTGGTTTTC
P547A	Forward	GCAGGACTGTGCAATTCTCGAGCACCACCAC
	Reverse	TCGAGAATTGCACAGTCCTGCTTGTTGCAGATCTG

Table S5: List of primer pairs for site-directed mutagenesis. All primer pairs are shown for site-directed mutagenesis of TSP1-TSR3 mutant constructs used in unfolding assays in Fig. 5 and for POFUT2 enzyme assays in Fig. S3. The lower-case letters denote which nucleotides were mutated from the wild type construct.

TSR3 WT Unmodified($^{13}C/^{15}N$ Heavy Labeled):

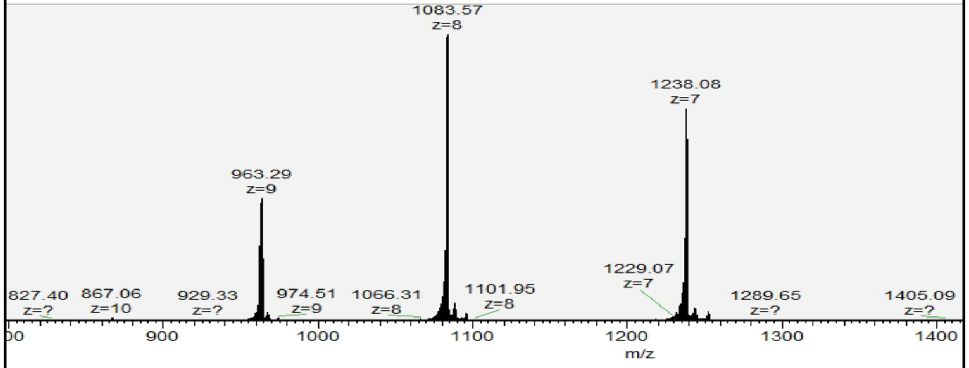

TSR3 WT-Fucose($^{13}C/^{15}N$ Heavy Labeled):

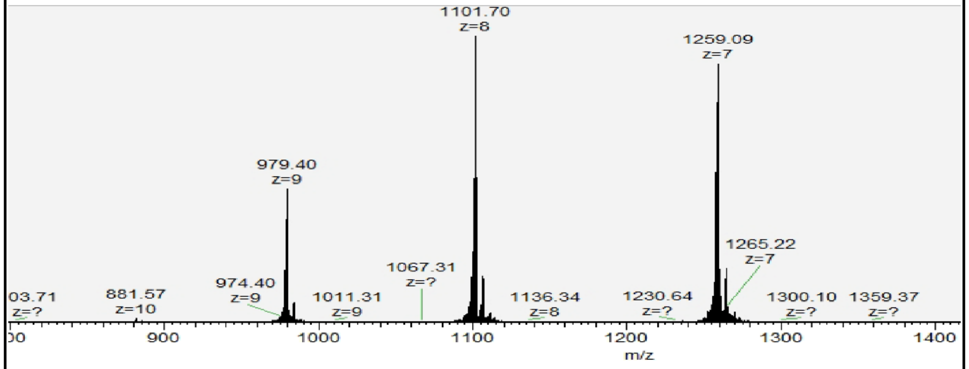

TSR3 WT-GlcFuc($^{13}C/^{15}N$ Heavy Labeled):

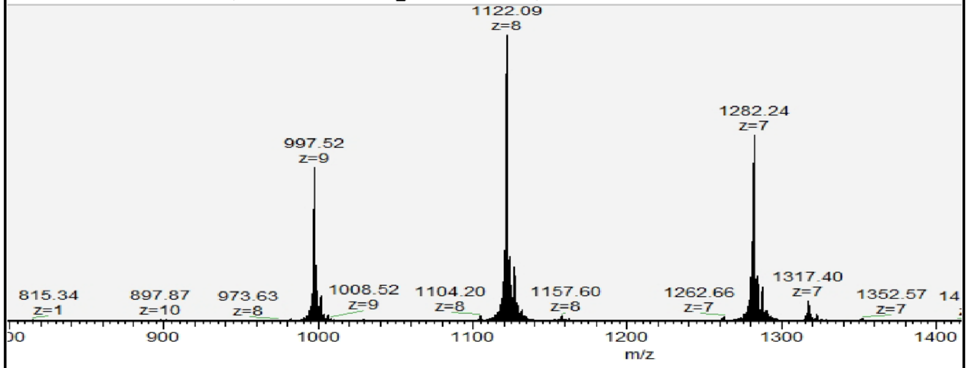

Charge State	Unmodified		Fuc		GlcFuc	
	$^{12}C/^{14}N$	$^{13}C/^{15}N$	$^{12}C/^{14}N$	$^{13}C/^{15}N$	$^{12}C/^{14}N$	$^{13}C/^{15}N$
6	1369.46	1445.93	1393.80	1470.27	1420.81	1497.28
7	1173.96	1239.51	1194.83	1260.37	1217.98	1283.53
8	1027.34	1084.70	1045.60	1102.95	1065.86	1123.21
9	913.304	964.29	929.53	980.51	947.54	998.52

Figure S1: NanoLC-MS analysis of unmodified and *O*-fucose modifications of $^{13}C/^{15}N$-labeled human TSP1-TSR3 for NMR studies. LC-MS analysis shows that the expressed TSR3 proteins used for NMR studies in Fig. 2A and Fig. 3A were nearly completely labeled with ^{13}C /^{15}N. A table under the spectra shows the theoretical mass-to-charge ratios for unlabeled ($^{12}C/^{14}N$) and heavy labeled (^{13}C /^{15}N) TSR3 for comparison. The ^{13}C /^{15}N labeled TSR3 was then modified *in vitro* with fucose monosaccharide and glucose-fucose disaccharide as described in Experimental Procedures. After modification and purification, samples were analyzed by nanoLC-MS to show that the labeled TSR3 was fully modified with *O*-fucose glycans. Spectra are shown for each glycoform (unmodified, fucose modified and glucose-fucose modified). Peaks of the most abundant charge states are shown, which were mainly charge states 7-9.

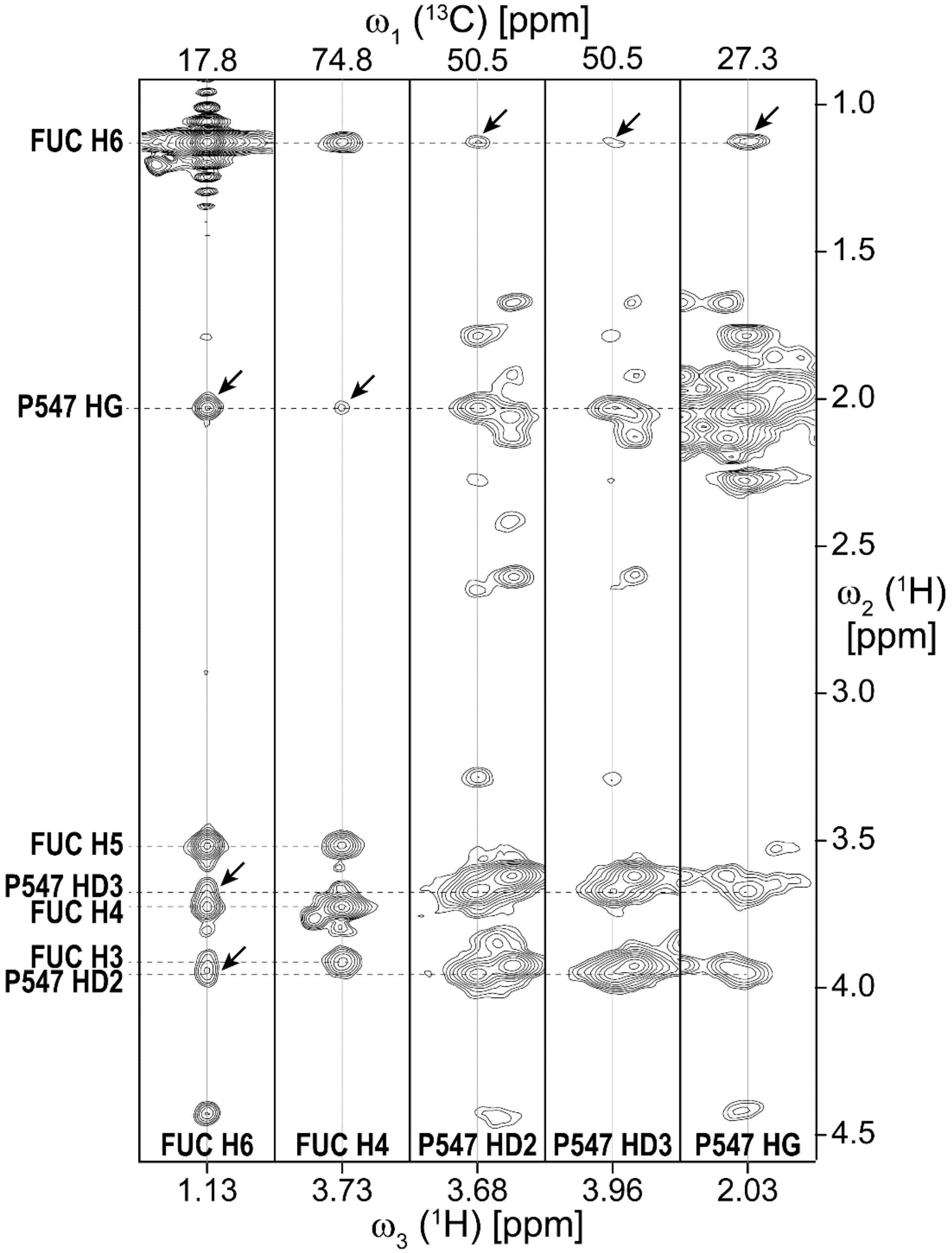

Figure S2: Strip plot of 3D ^{13}C-edited [^{1}H, ^{1}H] NOESY spectrum for selected resonances of *O*-fucose and Pro547 of TSR3-Fuc. Arrows indicate NOE cross-peaks between H4 and H6 (methyl) resonances of *O*-fucose, and HD2, HD3 and HG resonances of Pro547. In the *O*-fucose H6 strip these cross peaks overlap with H6-H4 and H6-H3 cross peaks. HD2 and HD3 resonance assignments are not stereospecific, HG represents both HG2 and HG3 spins due to chemical shift degeneracy.

A.

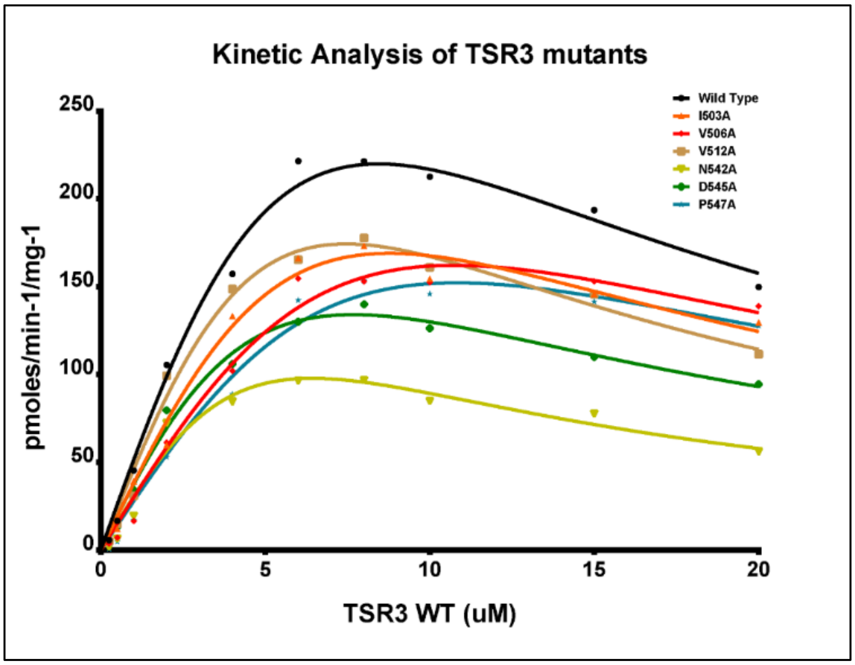

B.

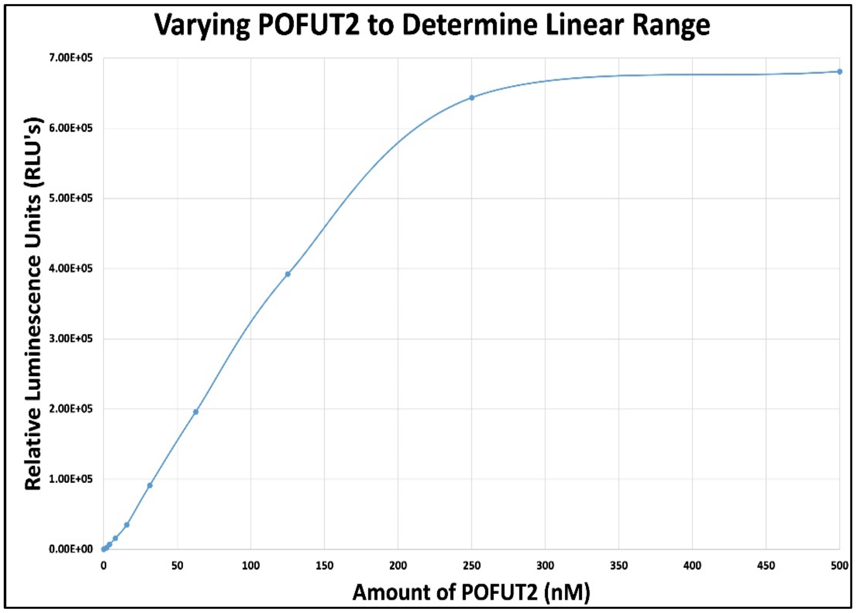

Figure S3: Analysis of wild type TSR3 and mutants as POFUT2 substrates. GDP-Glo assays (Promega®) were used to determine if TSR3 mutants were poor substrates for POFUT2 when compared to wild type. **(A)** Kinetic analysis showing that all TSR3 mutants are poorer substrates for POFUT2 than wild type. The curves were generated in Prism using a substrate saturation fit since either product or substrate inhibition was observed when using high concentrations of TSRs. **(B)** Linear range of POFUT2 to use for the assays. Once determining the linear range, 50 nM of POFUT2 was used for the data in panel A.

A: WT TSR3

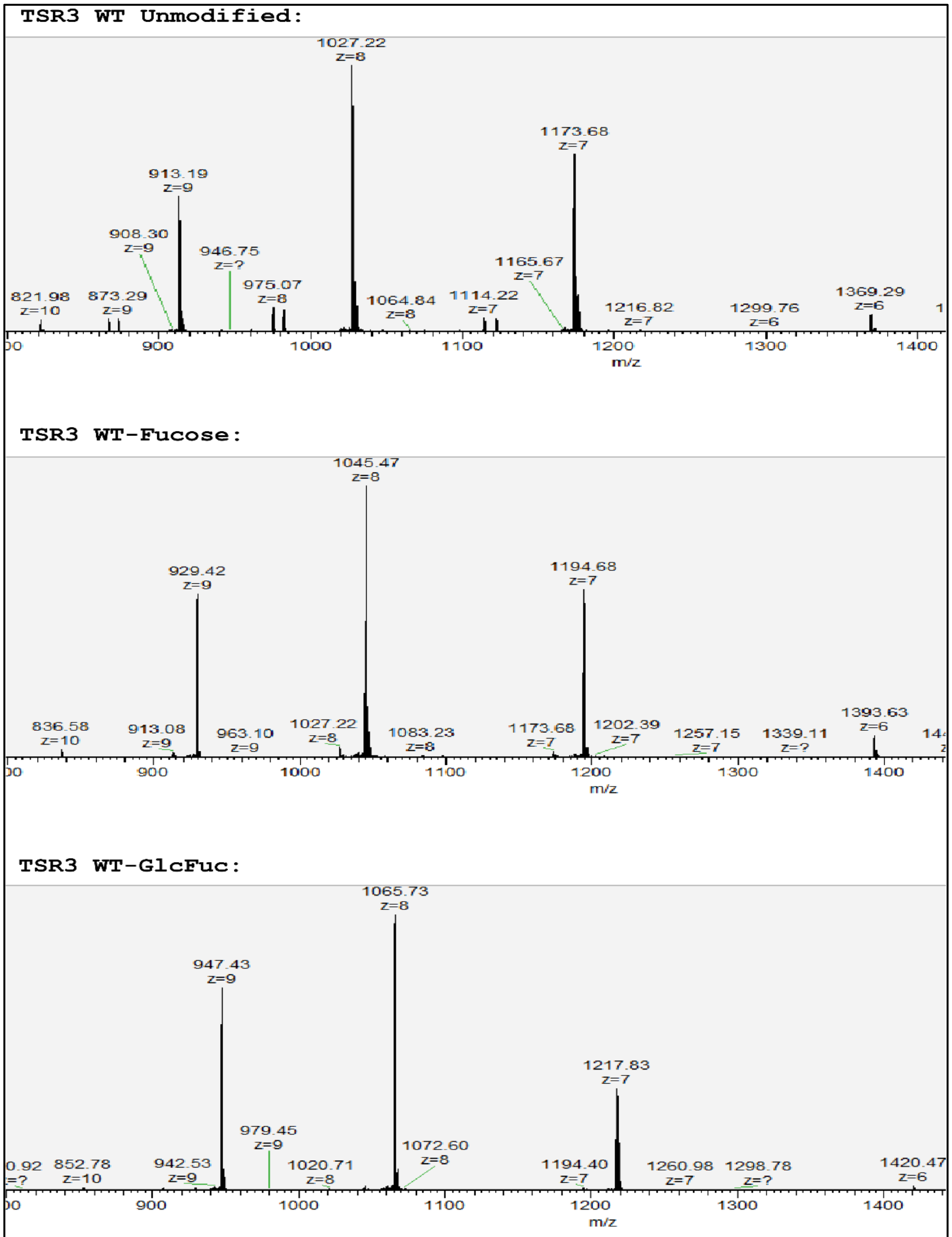

Charge State	Unmodified	Fuc	GlcFuc
6	1369.46	1393.80	1420.81
7	1173.96	1194.83	1217.98
8	1027.34	1045.60	1065.86
9	913.30	929.53	947.54

B: I503A mutant

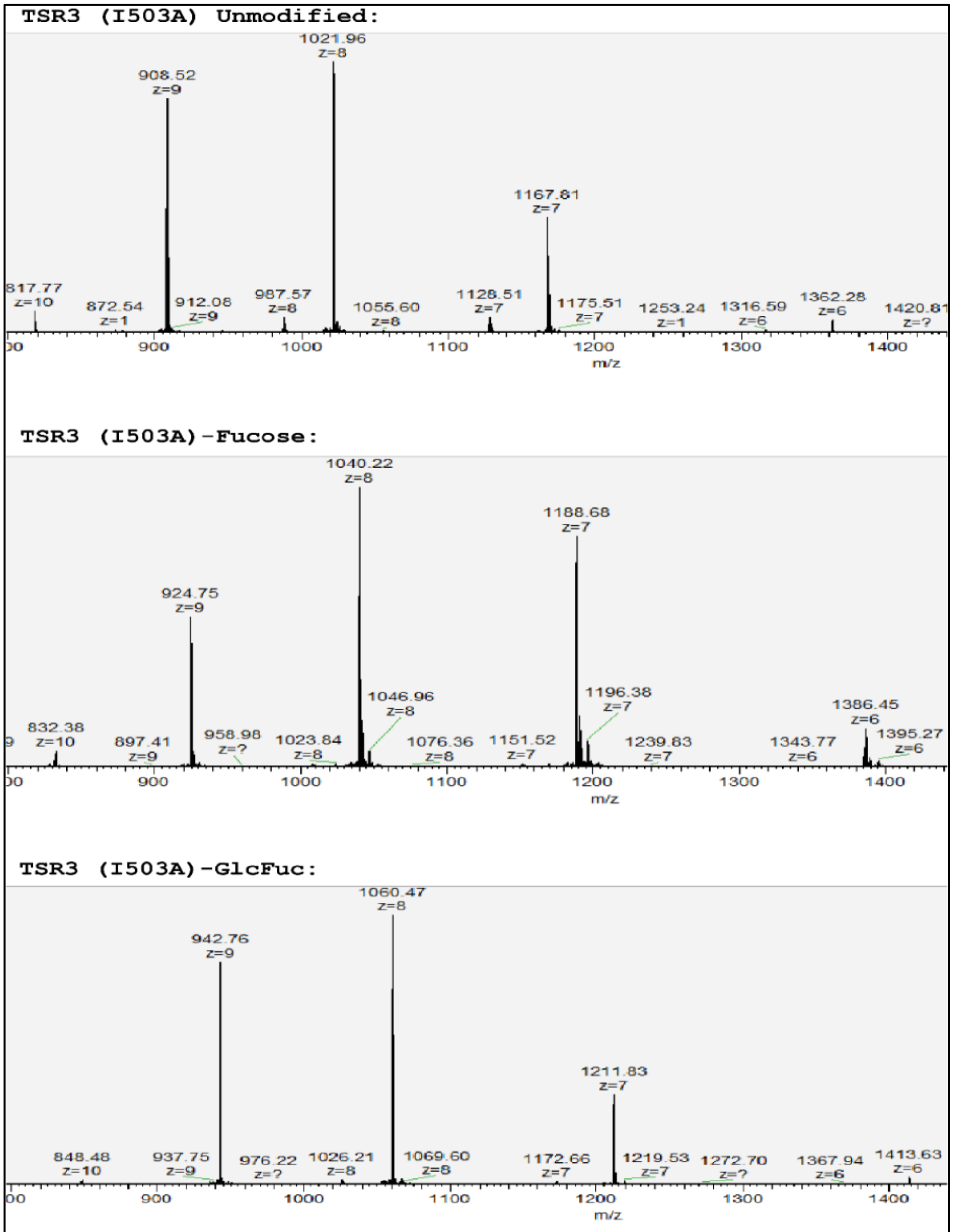

Charge State	Unmodified	Fuc	GlcFuc
6	1362.45	1386.79	1413.80
7	1167.96	1188.82	1211.97
8	1022.09	1040.34	1060.60
9	908.63	924.86	942.87

C: V506A mutant

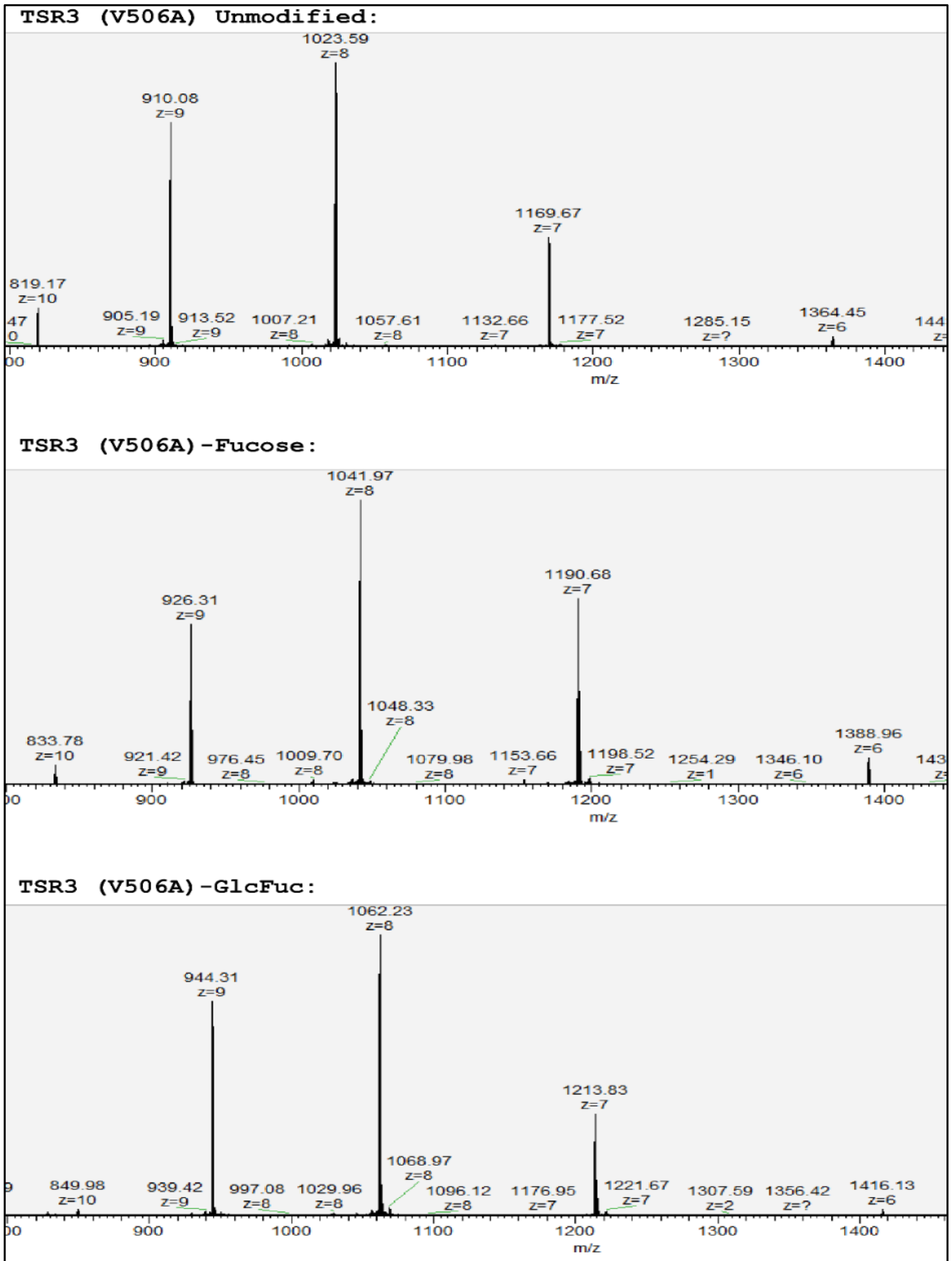

Charge State	Unmodified	Fuc	GlcFuc
6	1364.78	1389.13	1416.14
7	1169.96	1190.82	1213.97
8	1023.84	1042.10	1062.35
9	910.19	926.42	944.42

D: V512A mutant

TSR3 (V512A) Unmodified:

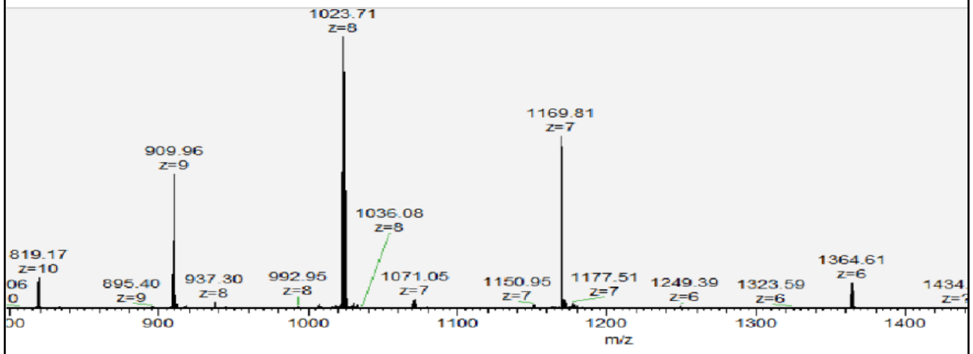

TSR3 (V512A)-Fucose:

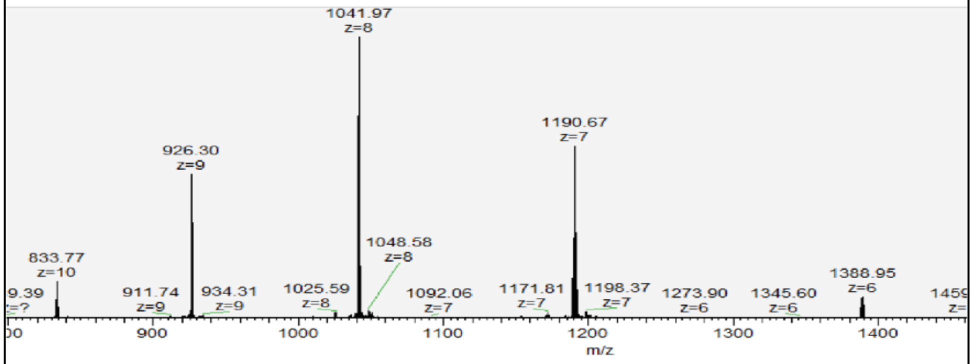

TSR3 (V512A)-GlcFuc:

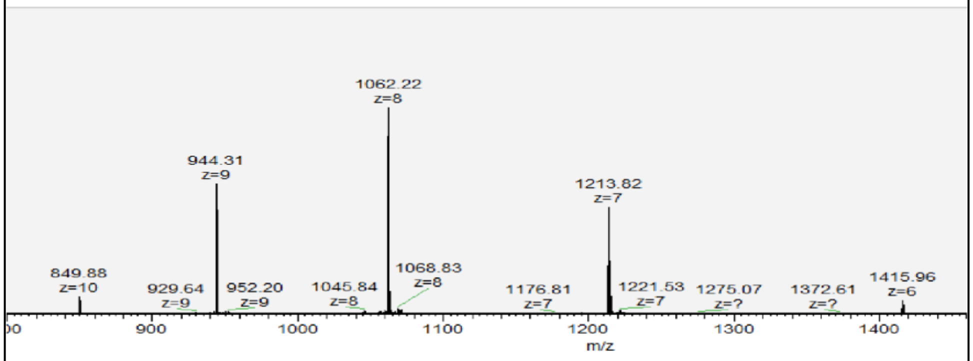

Charge State	Unmodified	Fuc	GlcFuc
6	1364.78	1389.13	1416.14
7	1169.96	1190.82	1213.97
8	1023.84	1042.10	1062.35
9	910.19	926.42	944.42

E: N542A mutant

TSR3 (N542A) Unmodified:

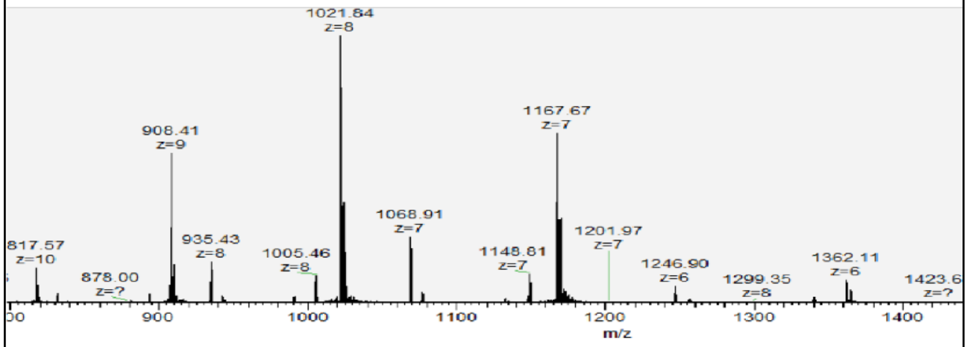

TSR3 (N542A)-Fucose:

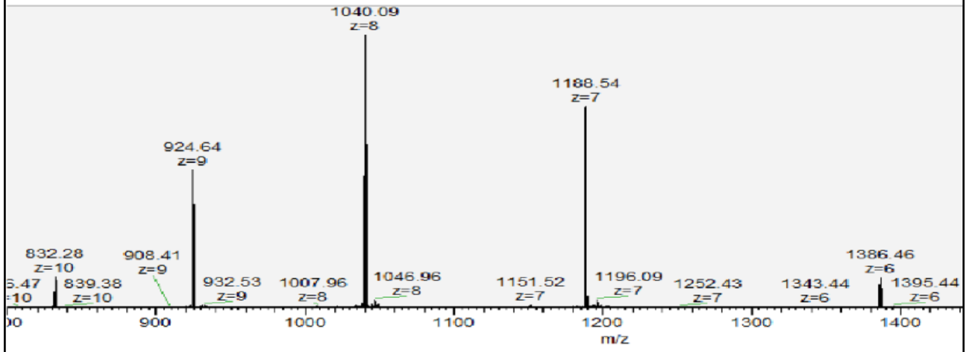

TSR3 (N542A)-GlcFuc:

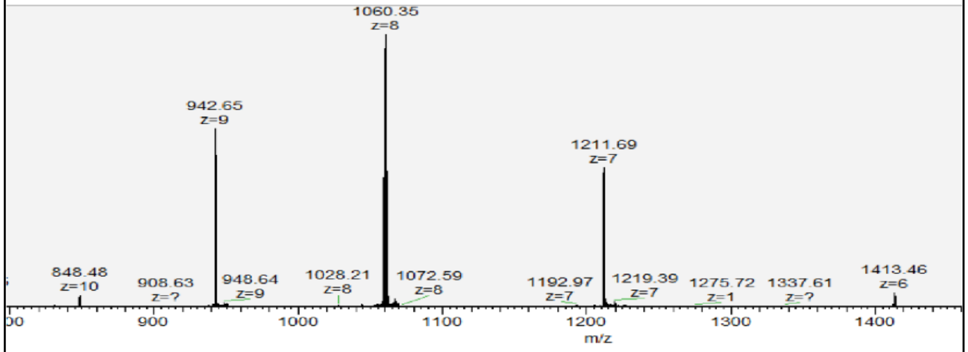

Charge State	Unmodified	Fuc	GlcFuc
6	1362.29	1386.63	1413.64
7	1167.82	1188.68	1211.83
8	1021.97	1040.22	1060.48
9	908.53	924.75	942.76

F: D545A mutant

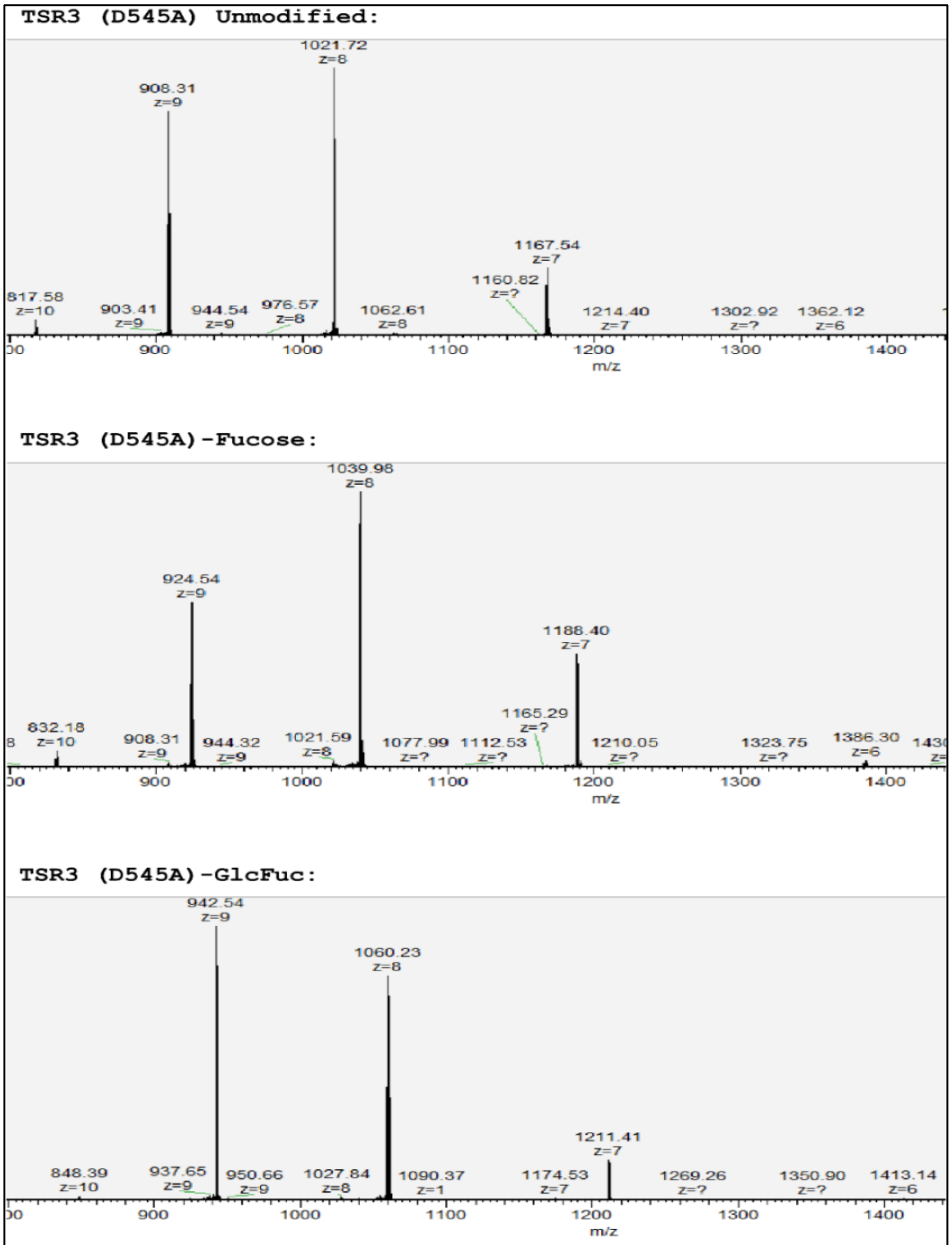

Charge State	Unmodified	Fuc	GlcFuc
6	1362.12	1386.47	1413.48
7	1167.68	1188.54	1211.69
8	1021.84	1040.10	1060.36
9	908.42	924.65	942.65

G: P547A mutant

TSR3 (P547A) Unmodified:

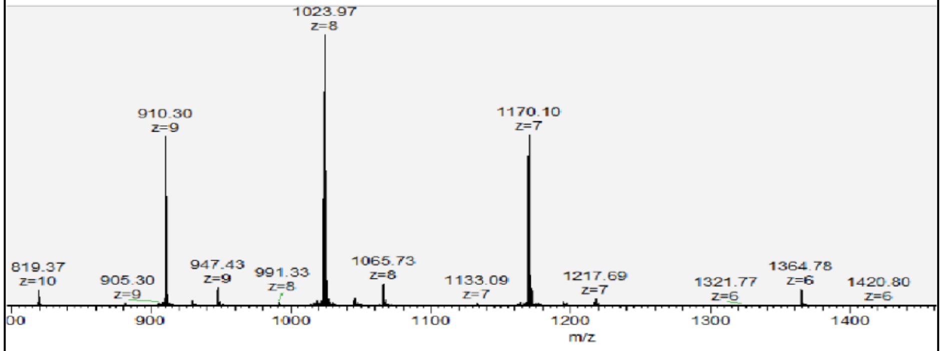

TSR3 (P547A)-Fucose:

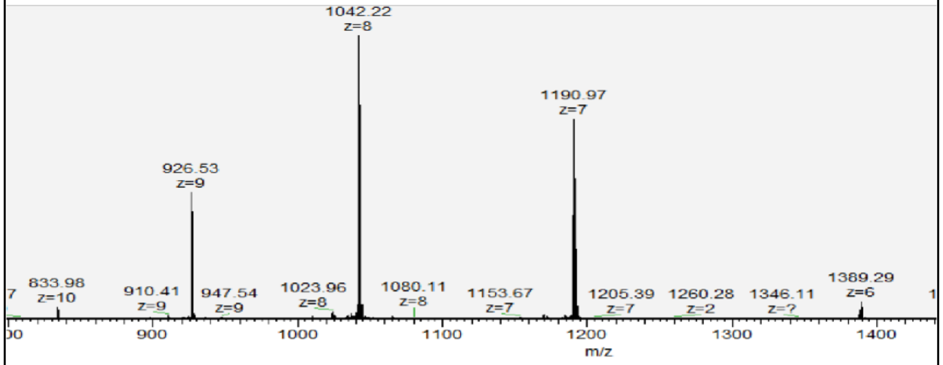

TSR3 (P547A)-GlcFuc:

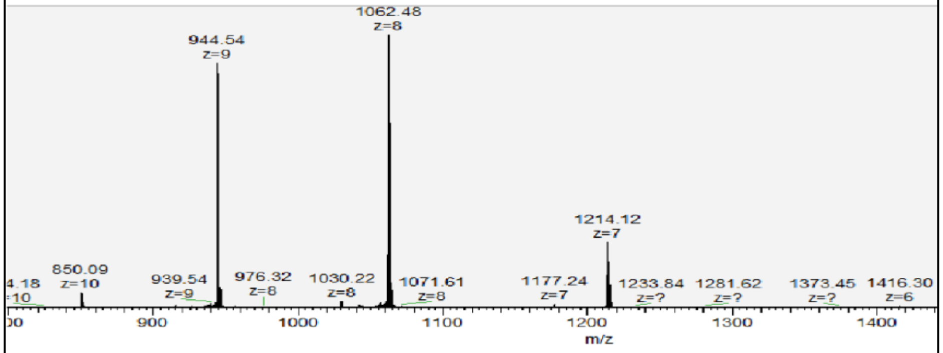

Charge State	Unmodified	Fuc	GlcFuc
6	1365.12	1389.46	1416.47
7	1170.25	1191.11	1214.26
8	1024.09	1042.35	1062.60
9	910.41	926.64	944.65

Figure S4: NanoLC-MS analysis of unmodified and *O*-fucose modifications of WT and mutant TSRs. Wild type TSP1-TSR3 and mutants were modified *in vitro* with fucose monosaccharide and glucose-fucose disaccharide for reductive unfolding assays shown in Fig. 5. After modification and purification, samples were analyzed by LC-MS to show that all TSRs were fully modified. For each construct (wild type and mutants), spectra are shown for each glycoform (unmodified, fucose modified and glucose-fucose modified). Peaks of the most abundant charge states are shown, which were mainly charge states 6-9. A table under each mutant spectra shows the theoretical mass-to-charge ratios for wild type and mutant TSR3. **(A)** WT; **(B)** I503A mutant; **(C)** V506A mutant; **(D)** V512A mutant; **(E)** N542A mutant; **(F)** D545A mutant; **(G)** P547A mutant.
